# Sleep fragmentation affects glymphatic system through the different expression of AQP4 in wild type and 5xFAD mouse models

**DOI:** 10.1186/s40478-022-01498-2

**Published:** 2023-01-18

**Authors:** Valeria Vasciaveo, Antonella Iadarola, Antonino Casile, Davide Dante, Giulia Morello, Lorenzo Minotta, Elena Tamagno, Alessandro Cicolin, Michela Guglielmotto

**Affiliations:** 1 Department of Neuroscience Rita Levi Montalcini, University of Torino, Via Cherasco 15, 10126 Turin, Italy; 2 Neuroscience Institute of Cavalieri Ottolenghi Foundation (NICO), University of Torino, Regione Gonzole 10, 10043 Orbassano, Turin, Italy; 3 Department of Neuroscience and Mental Health, AOU Città della Salute e della Scienza, Corso Bramante 88, 10126 Turin, Italy; 4 School of Pharmacy, Pharmacology Unit, University of Camerino, Via Madonna delle Carceri, 9, 62032 Camerino, MC Italy

**Keywords:** Alzheimer’s disease, Sleep fragmentation, Aquaporin-4 channel, Amyloid-β, p-tau, Neuroinflammation

## Abstract

Alzheimer’s disease (AD) is characterized by genetic and multifactorial risk factors. Many studies correlate AD to sleep disorders. In this study, we performed and validated a mouse model of AD and sleep fragmentation, which properly mimics a real condition of intermittent awakening. We noticed that sleep fragmentation induces a general acceleration of AD progression in 5xFAD mice, while in wild type mice it affects cognitive behaviors in particular learning and memory. Both these events may be correlated to aquaporin-4 (AQP4) modulation, a crucial player of the glymphatic system activity. In particular, sleep fragmentation differentially affects aquaporin-4 channel (AQP4) expression according to the stage of the disease, with an up-regulation in younger animals, while such change cannot be detected in older ones. Moreover, in wild type mice sleep fragmentation affects cognitive behaviors, in particular learning and memory, by compromising the glymphatic system through the decrease of AQP4. Nevertheless, an in-depth study is needed to better understand the mechanism by which AQP4 is modulated and whether it could be considered a risk factor for the disease development in wild type mice. If our hypotheses are going to be confirmed, AQP4 modulation may represent the convergence point between AD and sleep disorder pathogenic mechanisms.

**Supplementary Information:**

The online version contains supplementary material available at 10.1186/s40478-022-01498-2.

## Introduction

Alzheimer's disease (AD), the most common type of neurodegenerative disease, is generally characterized by memory loss, spatial learning disorder, and behavioral changes [1]. Only a small proportion of AD cases are familial, which is determined by gene mutations in the amyloid precursor protein (APP), presenilin 1 (PS1), and presenilin 2 (PS2) leading to increased production of amyloid-β (Aβ) [2, 3]. In sporadic cases, numerically more represented, not only the abnormal tau and Aβ accumulation, but also their affected clearance, seems to contribute to the pathogenesis of this disease. Mawuenyega et al. [4], measured and compared the Aβ production and clearance between AD patients and cognitively-normal individuals, and they demonstrated that the rates of Aβ clearance were slowed in AD patients, while rates of Aβ production were not altered. However, the cellular and molecular changes that impair Aβ clearance and render the aging brain vulnerable to Aβ plaque deposition remain unclear. It was recently reported that sleep is an important physiological process, during which extracellular metabolic wastes, such as amyloid and tau protein, are cleared via paravascular pathway [5–7]. In fact, the brain relies on the glymphatic clearance pathway to remove these waste materials [7]. In the aging brain, the impairment of glymphatic pathway function slows the clearance of interstitial Aβ, rendering the aging brain vulnerable to neurodegenerative disease.

Several authors have independently shown that glymphatic flux depends upon the expression and perivascular localization of the astroglial water channel aquaporin‐4 (AQP4) [5, 8, 9], although there are literature data that contradict this view [10]. Perivascular AQP4 supports rapid water movement between perivascular space and glial syncytium, thus forming a convective bulk flow of interstitial fluid (ISF). Animals lacking AQP4 exhibit slower CSF influx and less interstitial solute clearance (70% reduction) [6, 8, 11]. Moreover, the deletion of the AQP4 in APP/PS1 transgenic mice results in increased interstitial Ab plaque accumulation, cerebral amyloid angiopathy, as well as loss of synaptic protein and brain-derived neurotrophic factor in the hippocampus and cortex [12].

It is also known that the deletion of AQP4 effectively eliminates the circadian rhythm in glymphatic fluid transport. The high polarization of AQP4 in astrocytic end-feet is under the control of the circadian rhythm, and thus, modulates bulk fluid movement, CSF–ISF exchange, and solutes clearance [13]. In contrast, there is also evidence that astrocytes repress SCN neurons and regulate circadian timekeeping via glutamate signaling [14]. On these bases, astrocytes and AQP4 present a checkpoint for the functional glymphatic system during deep sleep, the slow wave sleep (SWS).

Changes in the timing and structure of sleep occur across the lifespan. Increased sleep fragmentation and reductions in SWS represent the hallmark signs of age-related changes in sleep [15]. The origin of sleep disturbances in AD is thought to be multifactorial. Degeneration of neural pathways that regulate sleep wake patterns and sleep architecture as well as somatic or psychiatric link between sleep characteristics and cognitive decline in the elderly have been suggested [16, 17]. Indeed, many researchers suggest a link between these two diseases, and interestingly, many of the disorders cited above are well known to be considered as risk factors for the development of AD. Sleep disturbances are not restricted to those with AD but are also prevalent in patients with mild cognitive impairment (MCI) [18]. This condition has a significant impact on patients and caregivers, and it is considered also a major risk factor for early institutionalization.

Clinical studies have confirmed that neurodegenerative pathogenesis begins more than 20 years prior to positive detection of extracellular mis‐folded protein deposits and symptoms of clinically evident cognitive decline [19]. The preclinical stage could therefore be much more important than the late stages for the development of effective interventions for neurodegenerative diseases in clinical practice. On these bases, in this work we analyzed the effect of sleep fragmentation in wild type and 5xFAD mouse models.

## Methods

### Animals

Two-months-old no carrier male mice (control mice) and 2- and 6-month-old male B6SJL-Tg(APPSwFlLon, PSEN1 ∗ M146L ∗ L286V)6799Vas/Mmjax (5xFAD) mice were used for a sleep fragmentation protocol. Experimental procedures involving the use of live animals have been carried out by the guidelines established by the European Community Directive 86/609/EEC (November 24, 1986), Italian Ministry of Health and the University of Turin institutional guidelines on animal welfare (law 116/92 on Care and Protection of living animals undergoing experimental or other scientific procedures; authorization number: 470/2021-PR). Moreover, the Ethical Committee of the University of Turin approved this type of study. The animals were maintained under 12-h light/dark cycles and were provided with water and food ‘‘ad libitum’’ (standard mouse chow 4RF25-GLP, Mucedola srl, Settimo Milanese, Italy). Specifically, all the procedures were carried out in order to minimize the pain and distress in the animals and we used the fewest number of animals required to obtain statistically significant data.

### Sleep fragmentation protocol

Two-month-old wild type (wt) (total mice = 22) and 5xFAD mice (total mice = 22) and 6-month-old 5xFAD mice (total mice = 8) were positioned on a time-controlled tilting platforms (Stuart Scientific Platform Rocker STR6) connected to a time relay (Mini Asymmetrical Cycle Timer, AC / DC 12-240V GRT8-S2, Regun) able to regulate their activation according to a pattern of 3 min OFF/10 s ON. The mice were divided into two groups: the first group (n = 11 for 2-months old and n = 4 for 6-months old mice) underwent sleep fragmentation for 30 days all day long (24 h), while the second one (n = 11 for 2-months old and n = 4 for 6-months old mice) was kept in cages under the same environmental conditions as fragmented mice, but in the absence of a time-controlled tilting platform, for the same length of time. In order to evaluate the effect of the protocol on sleep–wake cycle, an electroencephalography (EEG) and electromyographic (EMG) recording was performed on three animals per group (wild type n = 3 and 5xFAD n = 3) for 8 days (4 days in normal sleep conditions and 4 during sleep fragmentation). Only the EEG data from the last day were considered, as we preferred the day when the mouse was most likely to show adaptation to the chosen fragmentation system. Each recording was analyzed considering the 24-h day on the basis of the light/dark cycles imposed by the enclosure (8.00 a.m.–8.00 p.m.).

### Surgery for EEG registration

The electrodes used for recording the electroencephalographic signal were prepared by assembling an insulated ultra-thin stainless-steel wire (0.3 mm diameter, A-M Systems, Inc.) with a stainless-steel miniature screw (diameter 1.2 mm, P1 Technologies), soldered to a connector for the electronic circuitry. The recording electrodes were put in contact with the dura mater in order to obtain an ipsilateral fronto-parietal EEG signal (referential derivation). The frontal screws (one intended to recording and one to anchor the system) were positioned ± 1.2 mm from the interhemispheric fissure and + 1.2 from Bregma. The parietal screws (one recording and one used as common reference) were placed ± 1.2 mm from the interhemispheric fissure and + 1.2 from Lambda. A pair of insulated ultra-thin stainless-steel wire (0.3 mm diameter, A-M Systems, Inc.) was inserted in the posterior nuchal muscle to record the electromyographic (EMG) signal [20]. During the entire procedure of implantation of the electrodes for EEG and EMG recording, the animal was deeply anesthetized with 3% isoflurane (gaseous anesthetic for veterinary use), mixed with O_2_ (2 L/min) and N_2_O (1 L/min), and kept on a heated support to avoid hypothermia. The whole device was firmly attached to the skull by covering it with dental cement. At the end of the surgical procedure a subcutaneous dose of ketoprofen 10 mg/kg was administered. The mice underwent the sleep fragmentation protocol 10 days after surgery, in order to allow adequate recovery time and post-surgery adaptation.

### Acquisition of bioelectrical signals

To allow the mouse to have the greatest possible degree of freedom of movement, a structure has been developed consisting of a tilting arm capable of keeping the signal transmission cables suspended and allowing them to rotate, in such a way as to support the mouse in its movements. The EEG and EMG signals were transmitted with a cable connected to a rotating swivel commutator (SL2 + 3C/SB, P1 Technologies), used as an interface with the system responsible for the pre-amplification, amplification and A/D conversion of the signal (Grass Telefactor Comet AS40 Amplifier System for polysomnographic studies). The EEG and EMG were filtered (EEG: 0.3–35 Hz; EMG 10–70 Hz; Notch filter to discard the activity in the 50 Hz band) and sampled at 200 Hz for data storage.

### EEG data analysis

The signals acquired were manually analyzed using the Embla RemLogic-E software. The traces of the EEG and EMG data, displayed simultaneously on a 20-s time window, have been divided into mini-epochs lasting 4 s, and each of them has been assigned value labels: "W" for the waking epochs, "TNREM" for Non-rapid-eye-movement sleep and "TREM" for Rapid-eye-movement sleep. Wakefulness was scored when the EMG tone was high and EEG was at low amplitude with δ and θ frequency components; NREM epochs were scored when the EMG was lower than in W and EEG was at high voltage with prominent δ frequency components; REM epochs were characterized by muscle atonia at EMG and low voltage at the EEG with predominant θ frequency components [20]. In order to assign the relative value to an epoch, the epoch must be entirely occupied by the relative stage. In cases where two different stages coexist in an epoch, it is excluded from the analysis by the label "Not scored". The total sleep time (TST) was defined as the sum of the time spent in NREM and in REM sleep.

### Elevated plus maze (EPM)

The EPM test was used to assess anxiety‐like behavior. The EPM consisted of two parallel open arms (30 × 5 cm, surrounded by a 0.25 cm high border) and two parallel closed arms (30 × 5 cm, surrounded by 15 cm high walls). The four arms join to a central platform (5 × 5 cm). The apparatus was raised 45 cm above the floor and was illuminated by a soft light placed in a corner of the room. The mice were placed in the room test completely in the dark or with soft light 1 h before the beginning of the test. The test was initiated by placing the mouse (n = 11 per condition) on the central platform of the maze, facing one of the open arms, and leaving it free to move for 10 min. The behavior of the mouse was continuously recorded by a video camera placed above the apparatus, and then through the use of Ethovision XT software, the first 5 min were analyzed. The parameters analyzed during the test were: the frequency in open and closed arms (frequency entering in open and closed arms), the latency to enter in open arms [seconds employee to enter in open arms (s)], the cumulative duration in closed and open arms [time spent in the different arms (s)], the distance in the area [total distance traveled in the area (cm)], the velocity in the arena [maximum speed achieved in the area (cm/s)], the distance in the open and closed arms [distance in the different arms (cm)], and the velocity in the open and closed arms [maximum speed achieved in the different arms (cm/s)] [21].

### Open field test (OFT)

The open field (OF) test was used to assess the anxiety‐like behavior and spontaneous motor activity of all groups. OF consisted of a square arena (60 × 60 cm), with a dark floor divided into 36 squares (10 × 10 cm). The 20 squares near the walls of the apparatus constitute the peripheral zone of the apparatus (edge) and represent the protected zone of the field, the middle 16 squares represent the exposed zone of the field or the center of the arena. The mice were placed in the room test completely in the dark or with soft light 1 h before the beginning of the test. The test began with the animal (n = 11 per condition) being placed in a corner of the arena and allowed to move freely for 10 min. The mouse behavior was recorded using a camera positioned above the arena and the first 5 min were analyzed by using Ethovision XT software. The parameters analyzed during the test were: the frequency entering in the center and in the arena edges, the cumulative duration in the center and in the arena edges [time spent in the center and in the arena edges (s)], the distance in the arena [total distance traveled in the arena (cm)], the velocity in the arena [maximum speed achieved in the arena (cm/s)], the distance in the center and in the arena edges (cm), the velocity in the center and in the arena edges [maximum speed achieved in the center and arena edge (cm/s)], the frequency of protect and un-protect rearing, and the frequency of grooming [21].

### Novel object recognition (NOR) test

Mice were subjected to the NOR test to assess their object recognition and short-term working memory [22]. The mice were placed in the room test completely in the dark or with soft light 1 h before the beginning of the tes. The apparatus consisted of a small opaque plexiglass chamber with the following dimensions: 50 cm × 25 cm × 25 cm. Mice (n = 11 per condition) were acclimated to the apparatus for 5 min before starting the task. The training session consisted of placing a mouse in the apparatus containing two similar objects and allowing it to explore for 10 min. The test session was performed after 60 min (short-term memory) in the same apparatus, but two dissimilar objects by shape were present, a familiar and a novel one in which the animals were allowed to explore the objects for 5 min. Both phases of the test were carried out in the dark, and behaviors were recorded using an infrared camera placed over the apparatus. Behavioral parameters were analyzed by using Ethovision XT software and are as follows: the frequency entering in the new and old object zones; the cumulative duration in the new and old zones, meaning the total time spent in the different zones (sec); the frequency interaction with the new and old objects, ment as the frequency of the times the animal sniffs the objects; the frequency of protect and un-protect rearing; and the frequency and the cumulative durations of grooming (s). We considered also the Discrimination Index (DI), meaning the time spent exploring the novel object relative to the total time spent exploring both objects [(N_new _− N_old_)/(N_new_ + N_old_), where N_new_ represents the frequency of the interaction with the new object, while N_old_ with the old one]. The resulting score ranges from − 1 to + 1, when positive, the animal interacts more with the novel object, when negative with the old one. The interaction with the new object can also be expressed as a function of the Recognition Index (RI), which is the ratio of the amount of frequency exploring new objects over the total frequency exploring both objects [N_new_/(N_new_ + N_old_)] [23].

### Y-maze test

The Y-maze test was performed to measure spatial memory. The test occurs in a Y-shaped maze with three plastic arms at a 120° angle from each other. The mice were placed in the room test completely in the dark or with soft light 1 h before the beginning of the test. After an introduction to the center of the maze, the animal (n = 11 per condition) is allowed to freely explore the three arms. Over the course of multiple arm entries, the subject should show a tendency to enter in the less recently visited arm. The number of arm entries and the number of triads are recorded in order to calculate the percentage of an alternation. An entry occurs when all four limbs are within the arm. For the adaptation phase, the mice were placed into arm 1 out of 2 of a maze Y shaped made by black polyvinyl chloride panels for 5 min. After 1 h of rest, allowing re-consolidation phase of memory, we tested the mice’s spatial memory with all the arms open and exploration of the novel arm, for 10 min. For each session we measured different parameters, in such a way to evaluate spatial memory, anxiety-related and explorative behaviors of the tester mouse: the total distance traveled [distance traveled in the arena (cm)], the time spent in different arms (s), the frequency of entry in different arms, the latency to enter in the new arm (s), the frequency of protect rearing, the frequency of un-protect rearing, the frequency of grooming (n, the alternations (it is counted if the mouse enters all selected zones consecutively without repeated zone visits), the max alternations (it the total number of possible zone alternations and is calculated by taking the total number of visits and subtracting the number of selected zone minus, Alternation index = alternations/max alternations * 100), and the arm entries (i.e. the order in which the mouse enters in the numbered arms) [21].

### Immunofluorescence staining and microscopy

Briefly, the mice were anesthetized with a ketamine/xylazine mixture (80 mg/kg of ketamine and 10 mg/kg of xylazine) administered intraperitoneally. At this point the mice were first perfused with 0.9% NaCl and then with 4% paraformaldehyde (PFA). The drawn brains were left other 4 h in PFA 4% for a post-fixation and then placed in 30% sucrose. The brains were then cut to obtain 40 μm thick slices, then washed in PBS and incubated overnight with primary antibodies in PBS, Triton 2% and normal donkey serum 1.5% (017-000-121, Jackson ImmunoResearch). The primary antibodies used were: 6e10 (1:2000, 803001, BioLegend), AT8 (1:200, MN1020, Invitrogen), GFAP (1:3000, ab53554, Abcam), AQP4 (1:1500, HPA014784, Sigma Prestige), iba-1 (1:1000, 019-19741, Wako Chemicals), CD31 (1:500, 550300, BD Biosciences). The day after, the slices were washed again in PBS and incubated for 2 h with secondary antibodies: CY3 conjugated AffiniPure donkey anti-rabbit or anti-goat IgG (1:400, 711-165-152 and 705-165-147, Jackson ImmunoResearch), Alexa-Fluor488 conjugated AffiniPure donkey anti-mouse IgG (1:400, 715-545-151, Jackson ImmunoResearch), Alexa-Fluor647 conjugated AffiniPure donkey anti-rabbit IgG (1:400, 711-605-152, Jackson ImmunoResearch). For counterstaining, the brain sections were incubated with 4,6-diamidino-2-phenylindole (DAPI, 1:500, D9564, Sigma-Aldrich) and then washed with PBS. Finally, the slices were mounted on a glass slide and stored away from light and subsequently examined under the Leica TCS SP5 confocal laser scanning microscope (DM6000CS Leica) with 63x/1.40 oil objective, or the Zeiss Axioscan system Z.1 at a magnification of 10x (Plan-Apochromat, 10x/0.45 M27).

### Image analysis

Regarding the regional analysis of the immunostaining, the slices were acquired with Zeiss Axioscan microscope. Each considered brain region was not fully analyzed, but we used brain slices (at least 3 slices per mouse) which differ by a minimum interval of distance from Bregma (lateral septum − 1045:1420 mm; dentate gyrus, retrosplenial and motor-sensory cortex, thalamus, hypothalamus, and basolateral amygdala − 2255:− 1355 mm). All slices were compared to the Allen Mouse Brain Atlas (mouse.brain-map.org). The images were analyzed with Fiji (version 2.3.0/1.53q, Wayne Rasband, NIH, USA). The image analysis was carried out by measuring the percentage of pixels, after setting the same threshold for each different experiment for all regions of interest (ROIs). Each region has different sizes and since their dimensions can vary between slices, we used different ROIs (mean area for lateral septum ~ 65,941 μm^2^, retrosplenial cortex ~ 27,887 μm^2^, motor-sensory cortex ~ 198,789 μm^2^, dentate gyrus ~ 113,222 μm^2^, thalamus ~ 215,862 μm^2^, hypothalamus ~ 92,535 μm^2^, and basolateral amygdala ~ 37,553 μm^2^).

### Microglia morphology analysis

As regards microglia, for each region we acquired three images per mouse (n = 3 not fragmented, n = 3 fragmented) with confocal microscope 63x/1.40 oil objective. To have statistical significance, we randomly chose three cells from those not associated with Aβ plaques, for a total of 27 cells per condition in each region (189 total cells per condition). To examine microglia morphology and study the complexity of cell structure, we analyzed the number of endpoints, junctions, and branches using AnalyzeSkeleton2D/3D plugin of ImageJ [24, 25]. To the right of each iba1 image, the skeletons of the cropped cells are provided as a representation of the original image for each region analyzed.

### Western blot analysis

Total brain extracts were obtained from a 20% (w/v) mouse brain homogenate in RIPA buffer containing 20 mM Tris–HCl pH 7.4, 150 mM NaCl, 2 mM EGTA, 1 mM EDTA, 1% TritonTM-X-100, 0.5 mM PMSF and protease inhibitors and then centrifuged at 14.000*g* for 20 min at 4 °C to obtain soluble proteins. The protein content was determined using the Bradford assay. Lysates (20 μg) were run on 4–12% Tris–HCl gradient PAGE gel (Invitrogen) and then transferred to nitrocellulose blotting membrane (Invitrogen). The primary antibody, anti-AQP4 (1:10.000, HPA014784, Sigma Prestige), was incubated overnight. Peroxidase-conjugated secondary antibody (Biorad) was incubated for 1 h at room temperature (RT) and revealed with Luminata Forte Western substrate (WBLUF0100, Millipore). The correct protein loading was controlled normalizing with β-actin antibody.

### Statistical analysis

Statistical analyses were performed using GraphPad Prism version 4.0 (GraphPad software, San Diego). All data are representative of the results of at least three independent experiments. All values were presented as mean ± standard error (SEM). Means were compared by one-way analysis of variance (ANOVA). Adjusted *p* values were corrected for multiple testing using the Bonferroni post hoc test, where adjusted *p* value < 0.05 was defined to be significant. Regarding behavioral analysis, we used Fisher’s Least Significant Difference (LSD) as a post hoc test, considering a *p* value < 0.05 which indicated a statistical significance. As regards the EEG analysis, the hypnograms (sleep and wake periods during a day) obtained in normal conditions and during sleep fragmentation were visually compared in order to confirm the efficacy of the protocol.

## Results

### Validation of sleep fragmentation protocol through electroencephalography (EEG) recordings

**Fig. 1:**
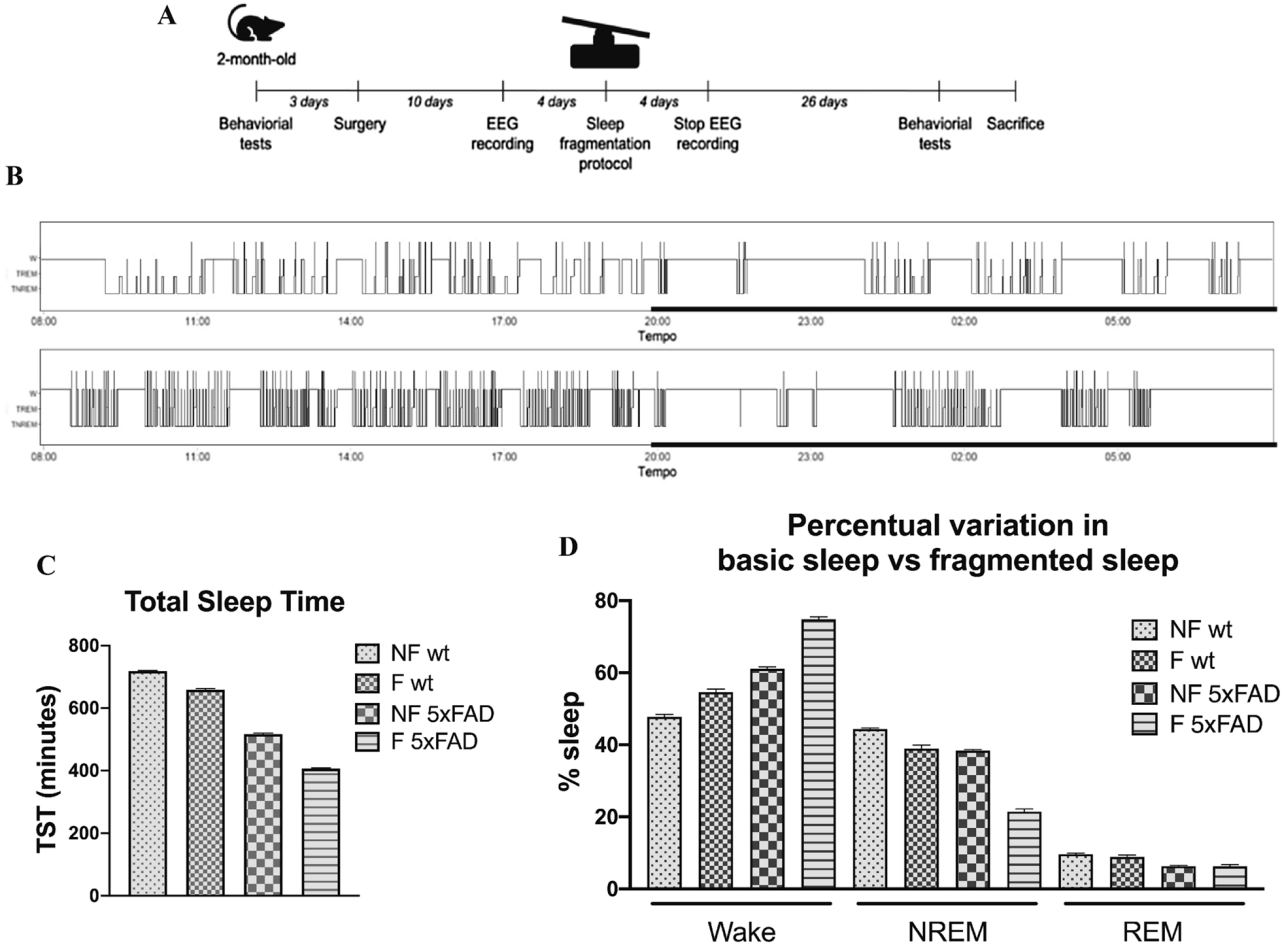
**A** Sleep fragmentation protocol time-line. **B** Typical hypnograms reporting sleep oscillations during baseline 24 h recording (top) and during fragmentation protocol (bottom). Thick lines indicate the dark period. **C** Total sleep time is represented as an average of the recordings. **D** The variation in the percentages relating to wakefulness, NREM sleep, and REM sleep. The two genotypes were compared for the three variables in basal and fragmentation conditions. *NF* not fragmented mice, *F* fragmented mice. The data are mean standard error of the mean (SEM), n = 3 per strain

As stated in methods section, the aim of our sleep fragmentation protocol was to achieve a chronic state of sleep fragmentation for 30 days, without significantly impairing the total amount of sleep (Fig. 1A). The hypnograms obtained in normal conditions and during sleep fragmentation periods were analyzed and as expected, both the wild type (wt) and the 5xFAD strains showed a significant increase of sleep/wake shifts (Fig. 1B). A mild decrease (10–25%) in the total sleep time (TST) during the 24 h recording was observed during sleep fragmentation period when compared to normal conditions (Fig. 1C), but such values are similar to normal data reported in literature for animals of the same age (the TST in *Mus musculus* strain BL6 ranges from 34.7 to 45.4% [26], 5% in C57BL/6N mice [27], for C57BL/6 strain approximately the 48.9% [28]). Although 5xFAD mice already display sleep alterations in comparison to wt animals, in both genotypes we noticed a decrease in NREM sleep during fragmentation and, conversely, an increase in the waking period, while REM sleep remains virtually unchanged, confirming our fragmentation protocol (Fig. 1D).

### Sleep fragmented 5xFAD mice show an accentuated anxious behavior analyzed by the elevated plus maze (EPM) and the open field test (OFT).

**Fig. 2:**
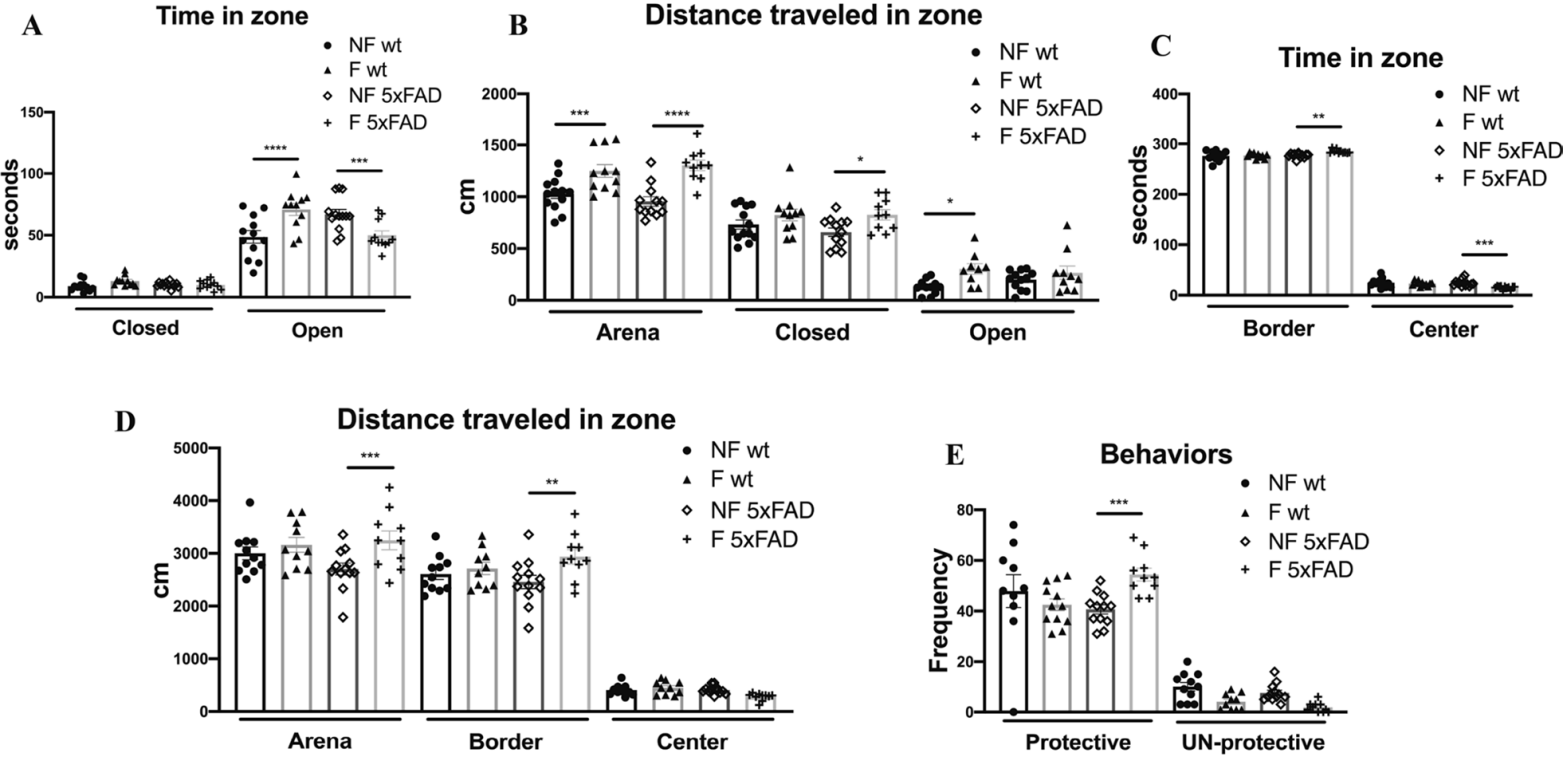
**A** Time spent in both closed and open arms during the EPM test. **B** Distance traveled in closed and open arms, and in the total arena during the EPM test. **C** Distance traveled in arena, border, and center in the OF test. **D** Time spent in both the border and the center of the arena during the OF test. **E** Behavioral activities during the OF test (protective and un-protective rearing). *NF* not fragmented mice, *F* fragmented mice. The data are mean standard error of the mean (SEM). Each data point represents an individual animal. *p < 0.05; **p < 0.01; ***p < 0.005; ****p < 0.0001 versus control by one-way ANOVA followed by Fisher’s LDS *post-hoc* test, n = 11 per condition

As shown in Fig. 2A, B, sleep fragmentation had different effects on anxious and hyperactive behavior in the EPM test. Fisher's multiple comparison test revealed increased anxiety in fragmented (F) 5xFAD mice by reducing the time spent in the open arms compared to not fragmented (NF) 5xFAD and F-wt mice (Fig. 2A), which in contrast spent more time in the open arms compared to their control group (NF-wt). Furthermore, sleep fragmentation increased anxiety in both strains of mice by increasing hyperactivity as shown in the total distance traveled in arena, which is greater in the closed arms than in the open arms (Fig. 2B). By contrast, F-wt mice showed a high motor activity even in the open arms compared to control (Fig. 2B). About the total time spent in the arena exploration, the F-5xFAD group showed an increase in the anxious behavior in the OFT that led the animals to spend more time on the edges of the apparatus and less in the center than all groups (Fig. 2C). In addition, the F-5xFAD group showed an increase in motor activity in the edges (Fig. 2D) and in the frequency of protective rearing (Fig. 2E), along with a reduction in motor activity in the center (Fig. 2C) and in the frequency of un-protective rearing (Fig. 2D) compared to the NF-5xFAD group.

### Sleep fragmentation impairs object recognition memory in the NOR test in both mouse strains

**Fig. 3:**
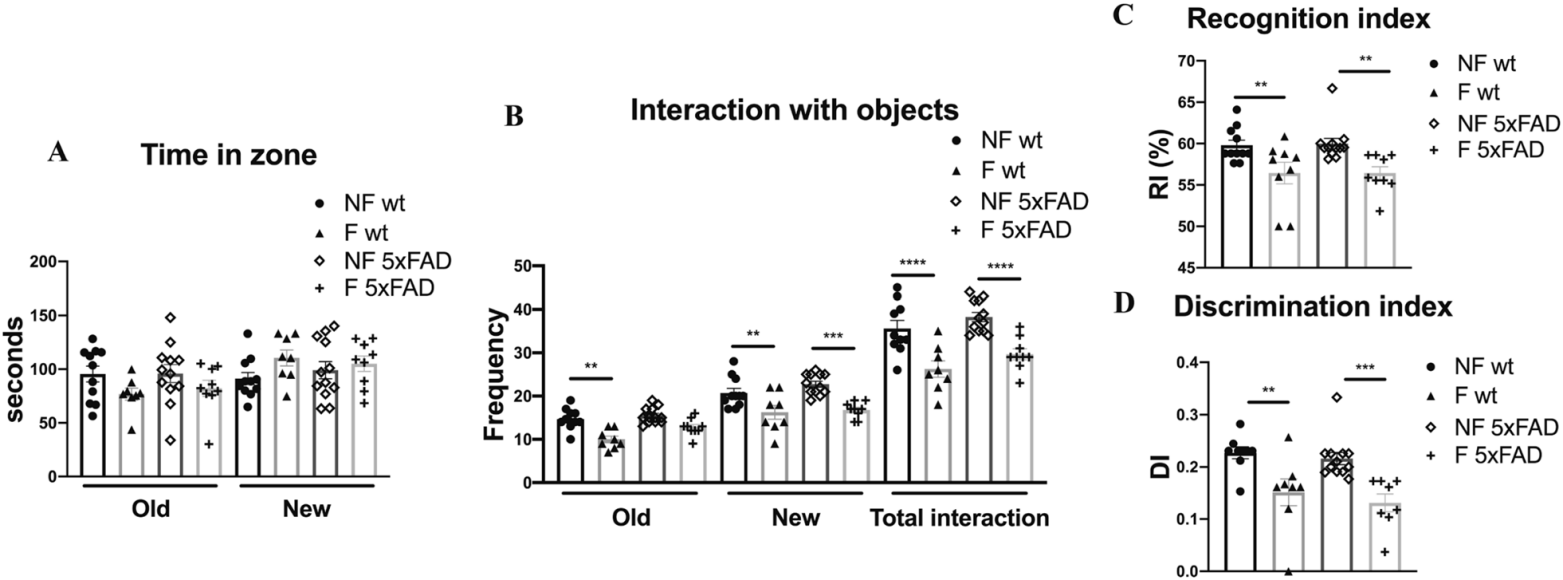
**A** Time spent near the old and the new objects. **B** Interaction with the old and the new objects and the total frequency of interaction with both objects. **C**, **D** Percentage of the discrimination and recognition indexes respectively. *NF* not fragmented mice, *F* fragmented mice. The data are mean standard error of the mean (SEM). Each data point represents an individual animal. *p < 0.05; **p < 0.01; ***p < 0.005; ****p < 0.0001 versus control by one-way ANOVA followed by Fisher’s LDS *post-hoc* test, n = 11 per condition

In this behavioral test, sleep disruption affected object recognition memory in both genotypes (Fig. 3A). Indeed, it reduced the interaction with the new and the old object (Fig. 3B), suggesting a compromised short-term memory. This finding confirmed with further analyses, in which the discrimination (Fig. 3C) and recognition indexes (Fig. 3D) are reduced in both strains after sleep fragmentation.

### Sleep fragmentation impairs spatial memory in the Y-maze test in 5xFAD mice

**Fig. 4:**
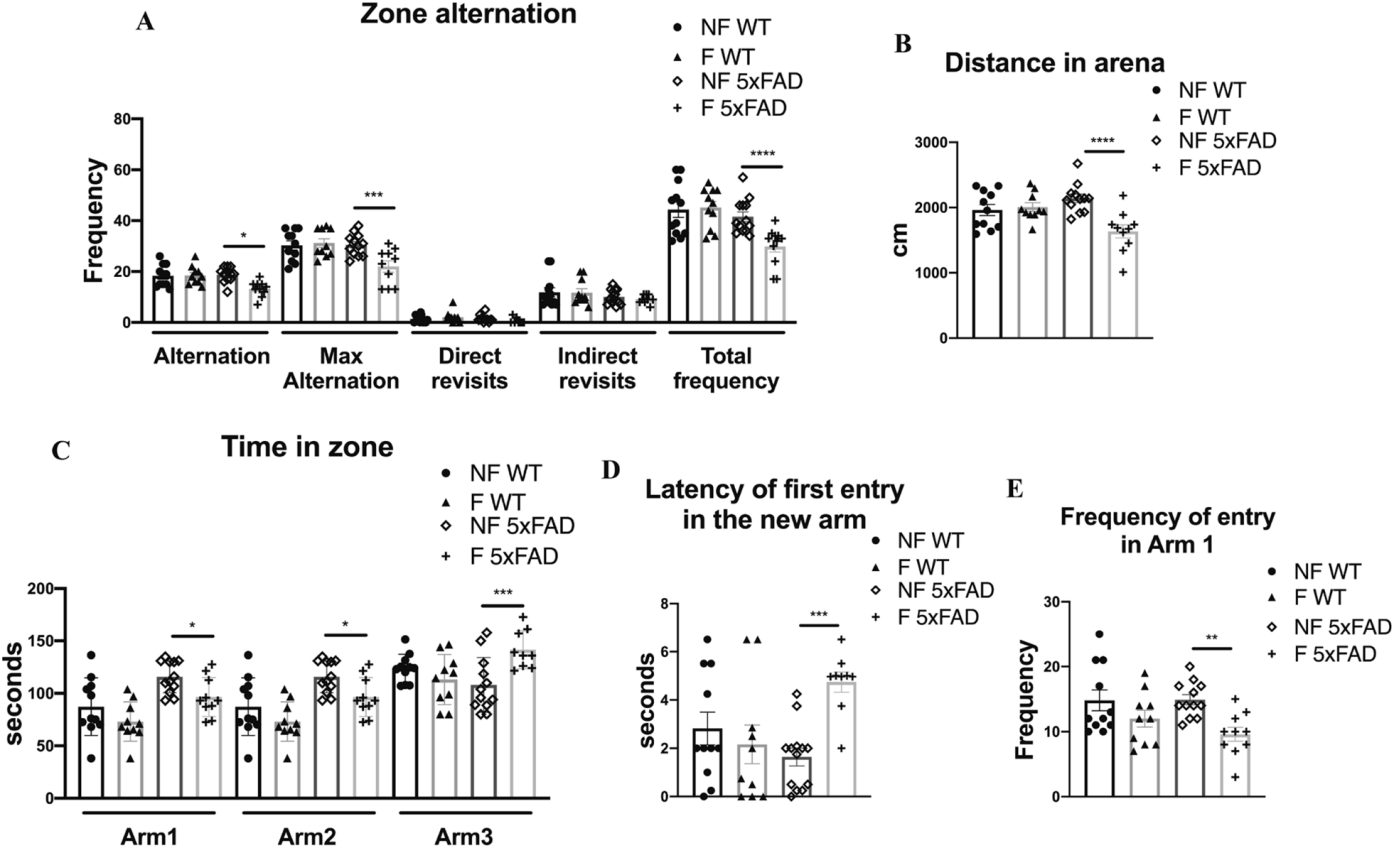
**A** Frequency of arm alternations. **B** Total distance traveled in the arena. **C** Time spent in the different arms. **D** Latency of the first entry in the new arm (arm 3). **E** Frequency of entry in the first arm (arm 1). *NF* not fragmented mice, *F* fragmented mice. *p < 0.05; **p < 0.01; ***p < 0.005; ****p < 0.0001

We evaluated the effects of sleep fragmentation on working and spatial memory by using the Y-maze test. The fragmented 5xFAD group showed a reduction in the percentage of alternation and maximal alternation entries in the arms, thus indicating memory impairment compared to all groups (Fig. 4A). The alternation percentage is correlated with the movement inside the apparatus (Fig. 4B) and with the time spent in the arms (Fig. 4C). Indeed, the F-5xFAD group traveled less distance in the apparatus (Fig. 4B), stopping longer in the new arm (Arm3; Fig. 4C) and taking more time to reach it respect all the other groups (Fig. 4D). In addition, memory impairment can be considered by evaluating the number of times the animal directly or indirectly reenters the previously visited arm. These two parameters correlate with the number of total frequencies of arm entries and thus with exploration. The F-5xFAD group showed a reduced total frequency of entry (Fig. 4A) compared to all groups, reducing the number of times it directly entered the first arm (Fig. 4E) and this may indicate a reduction in the exploratory ability. In the wt strain, sleep fragmentation did not affect any behavioral activity analyzed in this test.

### Sleep fragmentation accelerates AD progression by enhancing Aβ accumulation and inducing tau phosphorylation in 5xFAD mice

**Fig. 5:**
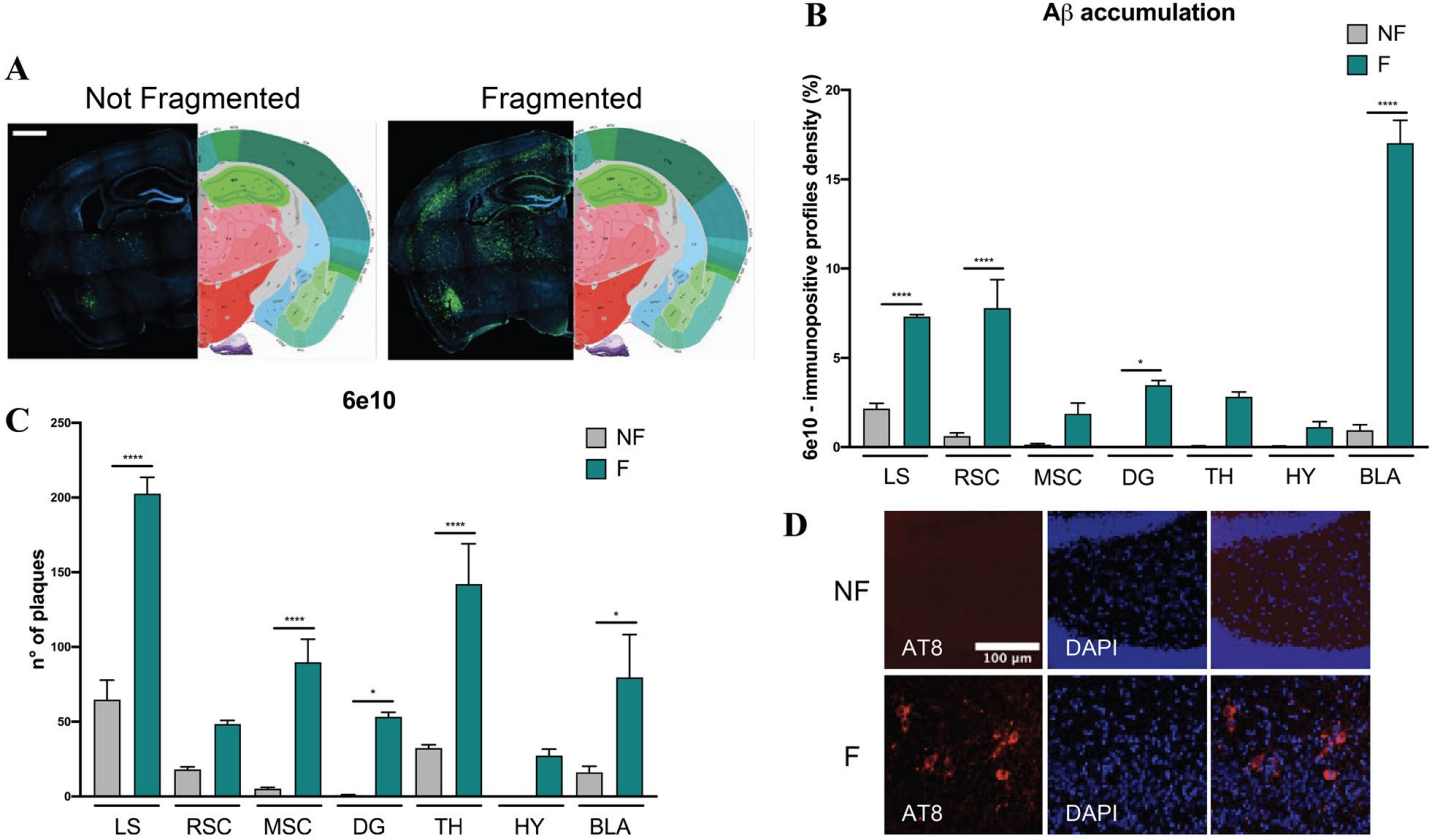
**A** Representative images of immunohistochemistry acquired with Axioscan microscope. Scale bar 1000 μm. Aβ plaques are labeled in green and the nuclei with DAPI in blue. **B** Histogram of Aβ plaques analyzed through the percentage of pixels, after the same threshold is set for all the region of interests. **C** Histogram of Aβ plaque number. **D** Representative images of immunohistochemistry of AT8. *NF* not fragmented mice, *F* fragmented mice, *LS* lateral septum, *RSC* retrosplenial cortex, *MSC* motor-sensory cortex, *DG* dentate gyrus, *TH* thalamus, *HY* hypothalamus, *BLA* basolateral amygdala. The data are mean standard error of the mean (SEM). Each data point represents an individual animal. *p < 0.05; ****p < 0.0001 versus control by one-way ANOVA followed by Bonferroni *post-hoc* test, n = 4 per condition

As for 5xFAD mice, this strain at 2 months of age already displays visible extracellular Aβ accumulation, thus we explored whether this accumulation could be more emphasized after the disruption of sleep. As shown in Fig. 5A, in fragmented 5xFAD mice compared to control, Aβ accumulation increased in both the cortexes and the dentate gyrus, as well as in all the other regions also involved in sleep regulation (Fig. 5B, C). Interestingly, Aβ accumulation increased also in the lateral septum (data not shown), a brain region which modulates cognitive processing in the cortex and hippocampus. We also observed an initiation of tau phosphorylation in the dentate gyrus in 5xFAD mice after sleep fragmentation compared to the not fragmented mice, where tau phosphorylation is not observed.

### Sleep fragmentation induces neuroinflammation by activating microglia and consequently astrocytes

**Fig. 6:**
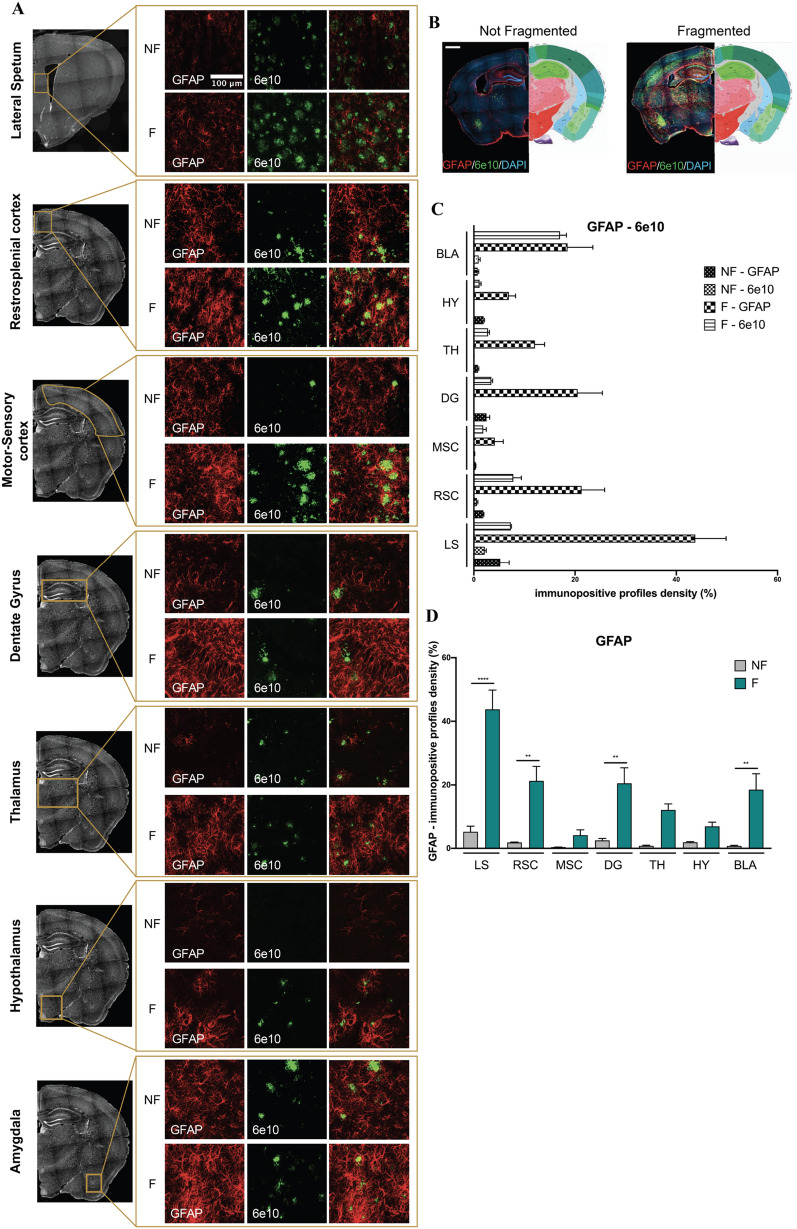
**A** Representative images of immunohistochemistry of GFAP and 6e10 antibodies in all the regions analyzed. **B** Representative images of immunohistochemistry of GFAP and 6e10 antibodies acquired with Axioscan microscope. Scale bar 1000 μm. **C** Histogram of GFAP and 6e10 shown together. **D** Histogram of GFAP density analyzed through the percentage of pixels, after the same threshold is set for all the region of interests. *NF* not fragmented mice, *F* fragmented mice, *LS* lateral septum, *RSC* retrosplenial cortex, *MSC* motor-sensory cortex, *DG* dentate gyrus, *TH* thalamus, *HY* hypothalamus, *BLA* basolateral amygdala. The data are mean standard error of the mean (SEM). Each data point represents an individual animal. **p < 0.01; ****p < 0.0001 versus control by ANOVA followed by Bonferroni *post-hoc* test, n = 4 per condition

**Fig. 7:**
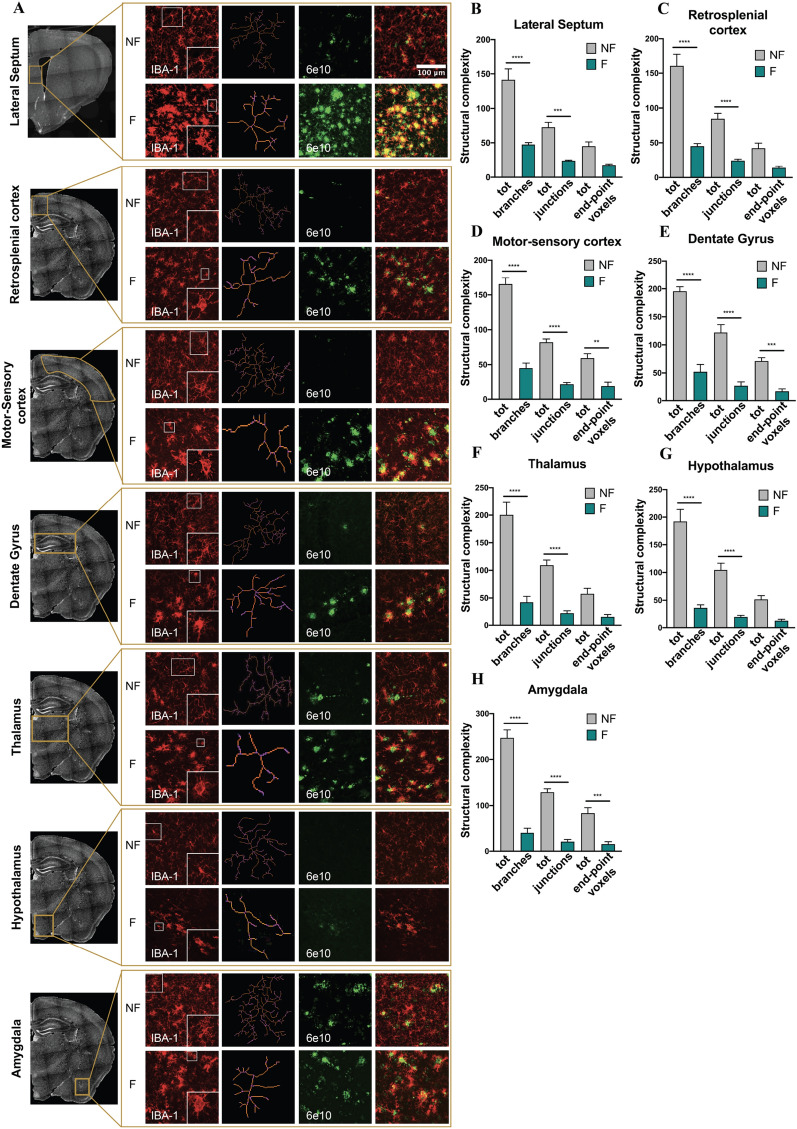
**A** Representative images of immunohistochemistry of iba-1 and 6e10 antibodies acquired with confocal microscope in all the regions analyzed. The process of analysis includes the skeletonization of microglia for the evaluation of cell complexity. A representation of skeletonized cells is shown on the right of the iba-1 images for each brain region. **B–H** Analysis of the structural complexity of microglia cells by using AnalyzeSkeleton (2D/3D) ImageJ plugin. *NF* not fragmented mice, *F* fragmented mice. The data are mean standard error of the mean (SEM). **p < 0.01; ***p < 0.005; ****p < 0.0001 versus control by ANOVA followed by Bonferroni *post-hoc* test, n = 3 per condition

Neuroinflammation is known to occur in AD pathology. To validate an activation of the neuroinflammation mediated by sleep disruption, we analyzed by immunofluorescence the density of astrocyte cells. Indeed, GFAP + signal increased in all the areas analyzed in F-5xFAD mice compared to control, thus indicating a possible astrogliosis (Fig. 6A–D). Interestingly, this signal well correlates with the increase of Aβ plaque accumulation (Fig. 6C). To confirm this result, we also investigated the activation of microglia, which is known to activate astrocytes by the release of immune factors. Here, we observed a major activation of microglia in F-5xFAD mice compared to control in all the brain areas analyzed (Fig. 7A). This activation is notable by analyzing the morphological complexity of microglia cells (Fig. 7B–H). By using Fiji software, we firstly skeletonized every cell taken in exam, and analyzed them by AnalyzeSkeleton(2D/3D) ImageJ plugin. In sleep fragmented mice, iba1 + cells are more activated by comparing the number of cell branches, branch junctions, and the voxel end-points which significantly decreased in most of the regions analyzed compared to control, in which microglia cells are less activated and consequently more ramified.

### Sleep fragmentation differently influences AQP4 expression according to the severity of the disease

**Fig. 8:**
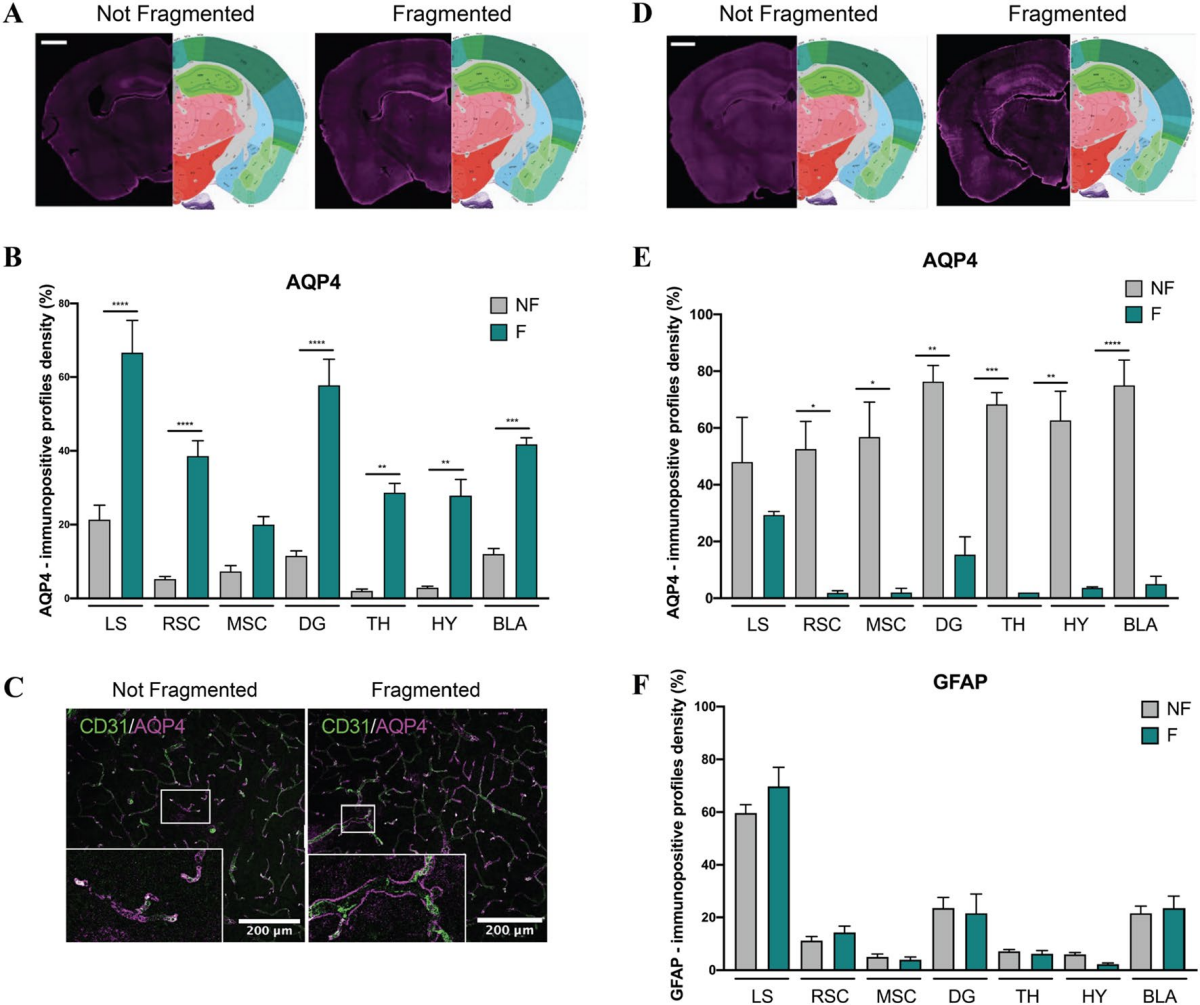
**A** Representative images of immunohistochemistry of AQP4 antibody acquired with Axioscan microscope in 2-months-old mice. Scale bar 1000 μm. **B** Histogram of AQP4 density in 2-months-old mice analyzed through the percentage of pixels, after the same threshold is set for all the region of interests. **C** Representative images of immunohistochemistry in 2-months-old mice of AQP4 and CD31, blood vessel marker, acquired with confocal microscope. **D** Representative images of immunohistochemistry of AQP4 antibody acquired with Axioscan microscope in 6-months-old mice. Scale bar 1000 μm. **E**, **F** Histograms of AQP4 and GFAP densities respectively in 6-months-old mice analyzed through the percentage of pixels, after the same threshold is set for all the region of interests. *NF* not fragmented mice, *F* fragmented mice, *LS* lateral septum, *RSC* retrosplenial cortex, *MSC* motor-sensory cortex, *DG* dentate gyrus, *TH* thalamus, *HY* hypothalamus, *BLA* basolateral amygdala. The data are mean standard error of the mean (SEM). Each data point represents an individual animal. *p < 0.05; **p < 0.01; ***p < 0.005; ****p < 0.0001 versus control by ANOVA followed by Bonferroni *post-hoc* test, n = 4 per condition

One of the clearance pathways of Aβ plaques is displayed by the glymphatic system, in particular by the activity of the AQP4 channel, located in the end-feet of astrocytes surrounding vessels. Since we observed an augmentation of Aβ accumulation mediated by sleep disruption, we investigated whether this clearance system is compromised. In 2-month-old 5xFAD mice, we observed an increase in the density of AQP4 + signal in all the brain areas involved (Fig. 8A, B). But despite the augmentation of Aβ plaques, we detected the AQP4 signal in the perivascular areas in both the control and the fragmented mice, thus indicating a possible functional channel activity (Fig. 8C). Interestingly, in older mice (6-month-old) we observed a decrease of AQP4 + signal (Fig. 8D, E), which could be due to a decrease in astrocyte cells. But when analyzing the density of astrocyte cells, we did not observe any significant change in the amount of GFAP + signal (Fig. 8F).

### Sleep disruption could affect the glymphatic system by decreasing AQP4 levels in wild type mice

**Fig. 9:**
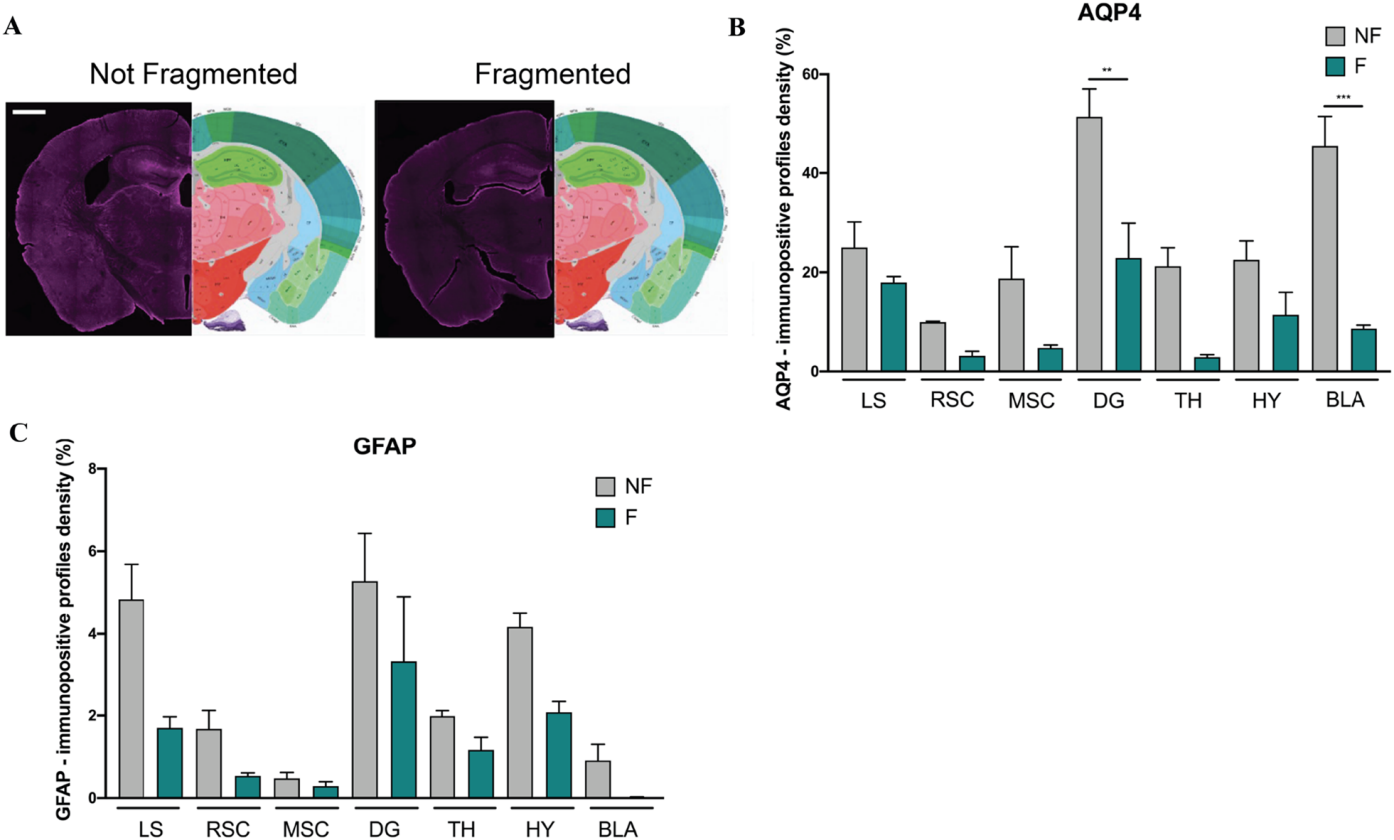
**A** Representative images of immunohistochemistry of AQP4 antibody acquired with Axioscan microscope in wild type mice. Scale bar 1000 μm. **B**, **C** Histograms of AQP4 and GFAP density respectively analyzed through the percentage of pixels, after the same threshold is set for all the region of interests. *NF* not fragmented mice, *F* fragmented mice, *LS* lateral septum, *RSC* retrosplenial cortex, *MSC* motor-sensory cortex, *DG* dentate gyrus, *TH* thalamus, *HY* hypothalamus, *BLA* basolateral amygdala. The data are mean standard error of the mean (SEM). Each data point represents an individual animal. **p < 0.01; ***p < 0.005 versus control by ANOVA followed by Bonferroni *post-hoc* test, n = 4 per condition

Wild type mice did not show any change in the hallmarks of AD pathology or in the activation of neuroinflammation (data not shown). This finding is linear with the behavioral analysis, in which sleep fragmentation did not significantly affect the behavior of the wild type strain, except for the object recognition memory in the NOR test. To clarify this result, we investigated whether the glymphatic system is affected by the fragmentation of sleep. Indeed, different articles report that a deficiency in the expression of the aquaporin-4 channel led to a defect in synaptic plasticity, learning, and memory [29, 30]. Intriguingly, we observed a significant decrease in the levels of AQP4+ signal after sleep fragmentation in the amygdala and the dentate gyrus (Fig. 9A, B), the two regions involved in learning, memory, and sleep regulation. Moreover, to better understand whether this data could correlate with a possible death of astrocyte cells, we also analyzed the density of GFAP-signal, which did not significantly change after sleep fragmentation (Fig. 9C).

## Discussion

Sleep is a vital process correlated to neural restoration and physiological maintenance across multiple systems [2, 31]. Indeed, this process is a highly conserved physiological phenomenon among mammals and it is important for multiple physiological activities, including cognitive abilities, immune function, and hormone release [32, 33]. During a normal night sleep, non-rapid eye movement (NREM) and rapid eye movement (REM) sleep alternately occur for 5–6 episodes in humans. Both sleep stages are important for learning and memory consolidation. Descriptive evidence implies that sleep may play a crucial role in optimal daytime cognitive abilities, attention, executive functioning, and memory [34, 35]. Changes in sleep are part of the normal aging process, with increased sleep fragmentation, nighttime awakenings and greater tendency to daytime sleep [36]. Intriguingly, disturbed sleep is associated with cognitive disorders [18]. Indeed, early observational data from institutionalized AD patients show worse nocturnal sleep among individuals with other severe forms of dementia [35], and these changes are already exacerbated in mild cognitive impairment (MCI) [37].

Our major aim was to investigate whether sleep fragmentation, which occurs in very common sleep disorders (e.g., insomnia, RLS, PLMD, and OSAS) could have a role in the pathogenesis and the progression of Alzheimer’s disease. To this end, the fragmentation protocol was applied to wild type and 5xFAD mouse strains. To appreciate the effect of sleep disruption at two different stages of aging and of AD, we studied very young (2-month-old) and young adult mice (6-month-old). Moreover, the use of control mice allowed to assess the consequences of a chronic fragmentation of sleep, not only in genetically predisposed animals, but also in mice in which no genetic mutation in favor of the disease is present.

Sleep fragmentation refers to poor quality of sleep due to multiple disruptions by extrinsic (e.g., noise) or intrinsic (e.g., apnea and limb movement) events. It consists of brief and frequent cortical arousal/microarousal followed by return to sleep, thus affecting the architecture of sleep [7]. The major causes of sleep fragmentation are obstructive sleep apnea, periodic limb movements, chronic pain, fibromyalgia, and gastroesophageal reflux disease (GERD) [38]. Short term sleep fragmentation leads to symptoms similar to sleep deprivation, mainly impaired attention, excessive sleepiness, emotional instability and exhaustion [39]. A possible explanation could be determined by an accelerated microglial ageing and activation [40].

In this study, the analysis of the hypnograms obtained in normal conditions and during sleep fragmentation periods demonstrated the validity of our experimental protocol, both for the wild type and the 5xFAD strains. The recordings shown a clear increase of the amount of sleep/wake shifts and a decreased NREM sleep respect wakefulness in mice subjected to a chronic sleep fragmentation. In this way we obtained an intermittent awakening, which is consistent with the sleeping pattern typical of aging, AD, and sleep disturbances. Healthy sleep is linked to the clearance of metabolic waste materials from the brain [41] and to the enhancement of cognitive functions, including the consolidation of memory [42, 43]. Conversely, sleep loss is associated to a different range of adverse effects, including deficits in cognitive activities [41, 44], dysregulation of circadian processes [45, 46] and impaired emotional function [47, 48].

Our goal was to identify sleep fragmentation as a risk factor for the onset of Alzheimer's disease and as a therapeutic target to control disease progression in AD patients. 5xFAD mice represent a severe Alzheimer’s disease mouse model [49], in which the parenchymal plaque load develops at only 2 months of age [50]. These mice have a high APP expression, which correlates with a high burden and an accelerated accumulation of the 42 amino acid species of amyloid-β (Aβ) [51]. We demonstrated that sleep fragmentation accelerates AD pathology. As shown in immunohistochemistry images, in young 5xFAD mice Aβ accumulation was already detectable in not fragmented mice, while we did not observe any evidence of tau phosphorylation. Contrarily, when we analyzed the immunochemistry of F-5xFAD animals, we found a strong increase of Aβ accumulation compared to control in all the areas involved not only in AD pathology, but also in sleep regulation. Interestingly, Aβ accumulation increased also in the lateral septum, a basal forebrain structure which modulates anxiety through its connection with the hippocampus [52, 53]. This confirms our results observed in the EPM and OFT, in which 5xFAD mice manifested an increase in anxious and hyperactive behavior (Fig. 2). In addition, we noticed a compromised spatial memory in 5xFAD strain after sleep fragmentation in the Y-maze test (Fig. 4). This observation is supported by several studies, which report that the lateral septum integrates the neuronal inputs from the hippocampus for the transformation of the cognitive map into actions [54]. Moreover, the retrosplenial cortex, which is one of the regions most affected by our experimental protocol in 5xFAD mice, is known to be another important area for spatial memory through its neuronal network with hippocampal processes [55]. A further interesting point, which could explain the behavioral differences observed in this study, is the presence of tau phosphorylation in the dentate gyrus in 5xFAD mice after sleep fragmentation compared to the not fragmented animals, in which tau phosphorylation is not detected. Indeed, we may assume that sleep fragmentation impairs object recognition memory (Fig. 3), a major component of declarative memory, which takes place in the hippocampus and it is modulated in the amygdala area [56, 57]. All the behavioral results are strongly supported by the fact that at the beginning of the protocol both wt and 5xFAD animals did not present any dissimilarity (Additional file 1: Table S1–S4). Moreover, the wt and 5xFAD not fragmented groups showed no significant variation both before and after the experimental protocol itself. This indicates that the statistical relevant differences in the cognitive capabilities that we observed in the fragmented animals are due to sleep fragmentation and not to the age or the severity of the disease (data not shown).

It is known that in peripheral tissues, lymphatic vessels return the excess of interstitial proteins to the general circulation for degradation in the liver [58]. In the brain, the same work is done by the glymphatic system, a glial-dependent waste clearance pathway dedicated to the drainage of soluble waste proteins and metabolic products [59]. According to many authors, a critical feature of the glymphatic system is its anatomical structure: it consists in a perivascular space (PVS), which is distinct from the highly complex and convoluted interstitial space of the brain parenchyma. The PVS surrounds the cerebral vascular system and it is lined by the end-feet of astrocytes plastered alongside the pericytes and endothelial cells that form the BBB [60, 61]. The first pioneering studies documented that soluble amyloid beta protein and tau oligomers are transported from the interstitial fluid (ISF) space and out of the brain via the glymphatic system [62, 63].

Acute changes in the geometry of the PVS through vasoconstriction and vasodilation have the potential to affect the movement of glymphatic fluid. Evidence for this hypothesis comes from mouse disease model of acute stroke, where ischemic spreading depolarization triggers the constriction of blood vessels, thus widening the PVS and enabling a rapid influx of CSF to the parenchyma [64]. Astrocytic swelling may have a role in these processes too. The primary evidence for astrocytic regulation of glymphatic fluid movement, beyond the spatial organization of the PVS, is that the astrocytic channel AQP4 facilitates glymphatic fluid transport [6, 8]. The expression of AQP4 is normally highly polarized toward the plasma membrane of the astrocytic end-feet facing the PVS. Mis-localization of AQP4 from astrocytic end-feet has been linked to glymphatic malfunction in multiple lines of work [8, 65–67]. Though AQP4 polarization toward the vascular end-feet constitutes a key regulatory mechanism, it is likely that astrocytes can alter glymphatic function by additional mechanisms. Glymphatic function is highly dependent on optimized perivascular spaces with low resistance to fluid flow. Nevertheless, very few studies have tested whether long-term remodeling of the shape, permeability, and patency of the PVS is actually linked to glymphatic dysfunction.

Based on the literature data, we wanted to investigate whether the increased production of amyloid-β, observed in 5xFAD mice subjected to fragmentation protocol, could be due to a failure of the glymphatic system. Since the regulation of AQP4 channel activity is one of the main ways through which the glymphatic system eliminates Aβ and tau aggregates [68], we decided to analyze the expression of this channel. However, in our analysis AQP4 resulted strongly augmented in F-5xFAD mice. In this case, we can speculate that this increase could not be related to channel activity because of the greater accumulation of Aβ in F-5xFAD animals. In fact, as reported above, mis-localization of AQP4 from astrocytic end-feet has been linked to the glymphatic malfunction in multiple lines of work. By contrast, we demonstrated that the distribution of AQP4 is near the perivascular areas in fragmented mice, similar to control (Fig. 8C). In addition, as shown in supplementary data (Additional file 1: Fig. S2), AQP4 still co-localized with GFAP+ cells, thus indicating that the increased AQP4 signal is determined by the expression level rather than its release in CSF, which is a typical sign of glymphatic failure [69]. This result is controversial, but a further remarkable point that could explain the dysfunctional increase of the expression of AQP4 is related to chronic pathologic changes, such as astrogliosis, which could also impair CSF influx, possibly through PVS alteration. Indeed, reactive gliosis is a common hallmark of neuropathology [70, 71]. We observed that sleep fragmentation increased GFAP-signal in 5xFAD mice, thus indicating a possible astrogliosis (Fig. 6). Since microglia is known to activate astrocytes through the release of those cytokines also involved in the pathological APP processing pathway [72, 73], we confirmed astrogliosis by our results through the analysis of the morphological complexity of microglia cells (Fig. 7). Many important aspects emerge from literature about the impairment of the glymphatic flux, such as the modulation of the perivascular space due to changes in both astrocytes and blood vessels. Intriguingly, the strong activation of astrocytes observed in F-5xFAD mice may cause the alteration of PVS size, as described by Mestre et al. [8], thus allowing the decrease of the glymphatic flux by the reduction of the PVS. Indeed, the primary evidence for astrocytic regulation of the glymphatic fluid movement, beyond the spatial organization of the PVS, is that AQP4 facilitates the glymphatic fluid transport [6]. Based on our results and literature data, we decided to further analyze AQP4 expression in older mice (6-month-old). Sleep fragmentation clearly decreased AQP4 signal in older 5xFAD animals without worsening astrogliosis (Fig. 8D–F), but with an additional impact on the clearance of Aβ. This result may be explained by Aβ load due to the advancement of the disease. Curiously, AQP4 signal decreased in wt mice after sleep fragmentation. Literature data show that a lack of AQP4 expression could cause a deficit in memory and learning [29, 30]. This analysis may be a crucial result since we observed that sleep disruption alters the recognition memory even in the wt strain (Fig. 3).

## Conclusions

In this article, we shed a new light on the study of AQP4 as one of the possible major markers for the study of sleep disorders in the pathogenesis of AD. In fact, we described an in vivo model in which AQP4 seems to be no longer functional and this is evident from the increased presence of Aβ plaques. This is also confirmed in older mice, in which the pathology is more advanced and AQP4 expression decreased, probably with a direct impact of AD and sleep fragmentation on the glymphatic system. We may interpret these data with the results obtained from wt animals. Indeed, this last result opens the possibility to further investigations, since we may suggest that the expression of AQP4 could represent a predictive marker for AD onset, already in middle age, thus providing an earlier diagnosis. Indeed, there is an urgent need to identify early biomarkers that determine which individuals are at greatest risk for AD development, motivated by at least two goals: (1) offering the chance for preventive measures, in the pre-disease onset phase, and (2) allowing nascent treatment intervention, early in the disease process.

## Supplementary Information

**Additional file 1**. **Table S1.** Differences in EPM data analyses between not fragmented (NF) and fragmented (F) animals before the beginning of the protocol. **Table S2.** Differences in OF data analyses between not fragmented (NF) and fragmented (F) animals before the beginning of the protocol. **Table S3.** Differences in NOR data analyses between not fragmented (NF) and fragmented (F) animals before the beginning of the protocol. **Table S4.** Differences in Y-maze data analyses between not fragmented (NF) and fragmented (F) animals before the beginning of the protocol. **Fig. S1.** AQP4 and its isoform expression. **Fig. S2.** AQP4 and GFAP colocalization analysis.

## Data Availability

The datasets used and/or analyzed during the current study available from the corresponding author on reasonable request.

## Abbreviations

AD: Alzheimer’s disease
AQP4: Aquaporin-4
APP: Amyloid precursor protein
PS1: Presenilin 1
PS2: Presenilin 2
Aβ: Amyloid-β
SWS: Slow wave sleep
OSA: Obstructive sleep apnea
RLS: Restless leg syndrome
PLMD: Periodic limb movement disorder
PTSD: Post-traumatic stress disorder
MCI: Mild cognitive impairment
EEG: Electroencephalography
EMG: Electromyography
REM: Rapid eye movement
NREM: Non-rapid eye movement
TST: Total sleep time
EPM: Elevated plus maze
OFT: Open field test
NOR: Novel object recognition
NF: Not fragmented
F: Fragmented
LS: Lateral septum
RSC: Retrosplenial Cortex
MC: Motor-Sensory cortex
DG: Dentate gyrus
TH: Thalamus
HY: Hypothalamus
BLA: Basolateral amygdala
GERD: Gastroesophageal reflux disease
PVS: Perivascular space
