# Supplementary material for: Sleep fragmentation affects glymphatic system through the different expression of AQP4 in wild type and 5xFAD mouse models
Source: Acta Neuropathol Commun. 2023 Jan 18;11:16. doi: 10.1186/s40478-022-01498-2 (PMC9850555; doi:10.1186/s40478-022-01498-2)
Supplement: Supplementary file 1 — Additional file 1. Table S1. Differences in EPM data analyses between not fragmented (NF) and fragmented (F) animals before the beginning of the protocol. Table S2. Differences in OF data analyses between not fragmented (NF) and fragmented (F) animals before the beginning of the protocol. Table S3. Differences in NOR data analyses between not fragmented (NF) and fragmented (F) animals before the beginning of the protocol. Table S4. Differences in Y-maze data analyses between not fragmented (NF) and fragmented (F) animals before the beginning of the protocol. Fig. S1. AQP4 and its isoform expression. Fig. S2. AQP4 and GFAP colocalization analysis. [file 40478_2022_1498_MOESM1_ESM.docx]

# Supplementary Materials

# Sleep fragmentation affects glymphatic system function through the different expression of AQP4 in wild type and 5xFAD mouse models

Valeria Vasciaveo^1,2^, Antonella Iadarola^3^, Antonino Casile^4^, Davide Dante^2^, Giulia Morello^1,2^, Lorenzo Minotta^2^, Elena Tamagno^1,2^, Alessandro Cicolin^1^, Michela Guglielmotto^1,2*^

^1^ Department of Neuroscience, University of Torino, Via Cherasco 15, 10126, Torino, Italy. ^2^ Neuroscience Institute of Cavalieri Ottolenghi Foundation (NICO), University of Torino, Regione Gonzole 10, 10043, Orbassano, Torino, Italy. ^3^ Department of Neuroscience and Mental Health, AOU Città della salute e della scienza, Corso Bramante 88, 10126, Torino, Italy. ^4^ University of Camerino, School of Pharmacy, Pharmacology Unit, Via Madonna delle Carceri, 9, 62032, Camerino (MC), Italy.

*To whom correspondance should be addressed:

Prof. Michela Guglielmotto,

Department of Neuroscience Rita Levi-Montalcini

Neuroscience Institute Cavalieri-Ottolenghi (NICO)

Regione Gonzole, 10

10043 Orbassano (To) – Italy

tel +39 011 6706604

fax +39 011 6705449

e-mail: michela.guglielmotto@unito.it

**Table S1: Differences in EPM data analyses between not fragmented (NF) and fragmented (F) animals before the beginning of the protocol**

| **ELEVATED PLUS MAZE** | | | | | | |
| --- | --- | --- | --- | --- | --- | --- |
| **PARAMETERS** | **NF wt** | **F wt** | **NF 5xFAD** | **F 5xFAD** | **ANOVA** | |
|  |  |  |  |  | **F** | **p-value** |
| **Frequency of entry in open arms (n°)** | 13,27 ± 1,50 | 12,73 ± 3,77 | 12,00 ± 1,41 | 11,80 ± 1,26 | 0,3 | 0,⁠824 |
| **Cumulative duration in open arms (s)** | 67,52 ± 15,06 | 64,08 ± 31,82 | 85,44 ± 13,42 | 68,56 ± 10,30 | 0,⁠65 | 0,59 |
| **Latency to enter in open arms (s)** | 8,03 ± 4,06 | 4,71 ± 10,42 | 3,43 ± 1,95 | 10,36 ± 3,45 | 1,05 | 0,⁠379 |
| **Frequency of entry in closed arms (n°)** | 21,45 ± 1,95 | 18,45 ± 3,83 | 17,92 ± 1,70 | 17,90 ± 1,16 | 1,12 | 0,⁠352 |
| **Cumulative duration in closed arms (s)** | 162,03 ± 12,75 | 162,15 ± 36,15 | 156,08 ± 8,55 | 159,33 ± 8,72 | 0,⁠38 | 0,767 |
| **Distance traveled in arena (cm)** | 1008,72 ± 61,31 | 1085,81 ± 222,00 | 1090,58 ± 83,37 | 985,24 ± 55,55 | 0,34 | 0,793 |
| **Velocity traveled in arena (cm/s)** | 3,36 ± 0,⁠20 | 3,62 ± 0,⁠74 | 3,64 ± 0,⁠28 | 3,52 ± 0,⁠25 | 0,18 | 0,908 |
| **Distance traveled in open arms (cm)** | 159,55 ± 39,74 | 160,85 ± 158,12 | 249,50 ± 38,51 | 115,50 ± 15,04 | 3,2 | **0,034** |
| **Velocity in open arms(cm/s)** | 2,34 ± 0,⁠23 | 3,12 ± 1,13 | 2,75 ± 0,⁠25 | 2,44 ± 0,⁠20 | 1,06 | 0,⁠378 |
| **Distance traveled in closed arms (cm)** | 716,85 ± 40,59 | 720,01 ± 119,57 | 713,01 ± 71,18 | 733,77 ± 60,52 | 0,⁠12 | 0,946 |
| **Velocity in closed arms (cm/s)** | 4,39 ± 0,⁠35 | 4,54 ± 1,14 | 4,88 ± 0,⁠38 | 4,50 ± 0,⁠31 | 0,44 | 0,946 |

All the parameters analyzed in the EPM test before the sleep fragmentation protocol begins. Only one significative change is relevant between NF wt and F wt and between NF 5xFAD and F 5xFAD. NF=not fragmented mice; F=fragmented mice

**Table S2: Differences in OF data analyses between not fragmented (NF) and fragmented (F) animals before the beginning of the protocol**

| **OPEN FIELD TEST** | | | | | | |
| --- | --- | --- | --- | --- | --- | --- |
| **PARAMETERS** | **NF wt** | **F wt** | **NF 5xFAD** | **F 5xFAD** | **ANOVA** | |
|  |  |  |  |  | **F** | **p-value** |
| **Frequency of entry in border (n°)** | 22,00 ± 2,44 | 22,80 ± 2,01 | 26,92 ± 3,42 | 23,30 ± 3,11 | 0,62 | 0,604 |
| **Cumulative duration in border (s)** | 275,88 ± 4,03 | 280,54 ± 1,82 | 274,04 ± 3,82 | 276,24 ± 2,43 | 0,69 | 0,565 |
| **Latency to enter in center (s)** | 16,33 ± 6,32 | 17,74 ± 5,85 | 26,11 ± 2,45 | 20,59 ± 6,54 | 1,92 | 0,142 |
| **Frequency of entry in center (n°)** | 21,09 ± 5,30 | 22,00 ± 2,00 | 26,00 ± 3,41 | 22,40 ± 3,17 | 0,6 | 0,619 |
| **Cumulative duration in center (s)** | 24,24 ± 4,04 | 19,62 ± 1,82 | 437,44 ± 3,82 | 23,92 ± 2,43 | 0,68 | 0,568 |
| **Distance traveled in arena (cm)** | 2662,59 ± 166,90 | 2990,75 ± 146,18 | 26,11 ± 163,37 | 3013,92 ± 148,11 | 1,49 | 0,233 |
| **Velocity traveled in arena (cm/s)** | 8,88 ± 0,⁠56 | 9,97 ± 0,⁠50 | 10,07 ± 0,⁠59 | 10,58 ± 0,65 | 1,54 | 0,22 |
| **Distance traveled border(cm)** | 2302,65 ± 133,41 | 2645,53 ± 146,18 | 2583,24 ± 132,45 | 2567,88 ± 78,80 | 1,5 | 0,23 |
| **Velocity traveled in border (cm/s)** | 8,36 ± 0,48 | 9,43 ± 0,50 | 9,46 ± 0,⁠52 | 10,00 ± 0,⁠59 | 1,71 | 0,181 |
| **Distance traveled in center (cm)** | 359,53 ± 51,78 | 327,47 ± 26,89 | 437,44 ± 63,80 | 449,83 ± 47,62 | 1,31 | 0,284 |
| **Velocity traveled in center (cm/s)** | 16,21 ± 1,51 | 17,63 ± 0,⁠80 | 17,40 ± 1,48 | 18,99 ± 1,69 | 0,62 | 0,605 |
| **Frequency of protect rearing (n°)** | 35,64 ± 2,53 | 39,50 ± 2,80 | 41,67 ± 5,08 | 34,90 ± 1,88 | 0,86 | 0,469 |
| **Frequency of un-protect rearing (n°)** | 12,36 ± 3,31 | 12,40 ± 2,84 | 7,75 ± 1,34 | 5,10 ± 1,27 | 2,27 | 0,096 |
| **Frequency of grooming (n°)** | 8,45 ± 1,72 | 5,50 ± 1,16 | 5,25 ± 1,20 | 6,50 ± 0,⁠78 | 1,32 | 0,28 |
| **Cumuative duration of grooming (s)** | 28,28 ± 7,65 | 33,09 ± 14,62 | 15,63 ± 5,59 | 17,09 ± 2,64 | 0,99 | 0,406 |

All the parameters analyzed in the OF test before the sleep fragmentation protocol begins. No significative change is relevant between NF wt and F wt and between NF 5xFAD and F 5xFAD. NF=not fragmented mice; F=fragmented mice

**Table S3: Differences in NOR data analyses between not fragmented (NF) and fragmented (F) animals before the beginning of the protocol.**

| **NOVEL OBJECT RECOGNITION** | | | | | | |
| --- | --- | --- | --- | --- | --- | --- |
| **PARAMETERS** | **NF wt** | **F wt** | **NF 5xFAD** | **F 5xFAD** | **ANOVA** | |
|  |  |  |  |  | **F** | **p-value** |
| **Frequency in new object zone (n°)** | 16,30 ± 1,37 | 20,11 ± 1,81 | 17,67 ± 1,75 | 17,60 ± 2,02 | 1,05 | 0,382 |
| **Cumulative duration in new object zone (s)** | 101,01 ± 11,03 | 96,25 ± 7,09 | 86,74 ± 7,51 | 78,88 ± 5,60 | 1,89 | 0,148 |
| **Frequency in old object zone (n°)** | 15,50 ± 1,43 | 19,00 ± 1,60 | 18,58 ± 1,49 | 20,60 ± 2,53 | 60,28 | **0,⁠000** |
| **Cumulative duration in old object zone (s)** | 91,80 ± 5,77 | 88,46 ± 9,61 | 96,32 ± 6,26 | 92,37 ± 8,32 | 86,01 | **0,⁠000** |
| **Frequency interaction new object (n°)** | 19,10 ± 1,32 | 17,88 ± 1,05 | 22,83 ± 1,95 | 23,00 ± 0,⁠91 | 3,39 | **0,⁠027** |
| **Frequency interaction old object (n°)** | 13,40 ± 0,⁠75 | 11,38 ± 0,⁠99 | 16,67 ± 1,29 | 15,89 ± 0,⁠86 | 5,09 | **0,⁠005** |
| **Frequency total interaction (n°)** | 32,50 ± 1,26 | 29,25 ± 1,90 | 39,50 ± 2,45 | 38,67 ± 1,65 | 6,47 | **0,⁠001** |
| **Discrimination Index (DI)** | 0,⁠17 ± 0,⁠05 | 0,⁠23 ± 0,⁠03 | 0,⁠15 ± 0,⁠05 | 0,⁠19 ± 0,⁠02 | 0,⁠83 | 0,⁠486 |
| **Recognition Index (RI)** | 58,43 ± 2,65 | 61,38 ± 1,43 | 57,34 ± 2,31 | 59,25 ± 0,⁠93 | 0,⁠82 | 0,⁠488 |
| **Frequency protect rearing (n°)** | 36,70 ± 0,⁠10 | 30,12 ± 3,97 | 32,75 ± 2,93 | 30,00 ± 2,56 | 0,⁠80 | 0,⁠502 |
| **Frequency un-protect rearing (n°)** | 3,00 ± 5,77 | 2,38 ± 0,⁠83 | 2,00 ± 0,⁠54 | 2,56 ± 0,⁠72 | 0,⁠19 | 0,⁠905 |
| **Frequency grooming (n°)** | 1,10 ± 0,⁠10 | 1,38 ± 0,⁠16 | 2,08 ± 0,⁠36 | 2,11 ± 0,⁠18 | 4,96 | **0,⁠005** |
| **Cumulative duration grooming (s)** | 10,50 ± 2,10 | 14,09 ± 3,69 | 17,54 ± 3,86 | 19,60 ± 3,02 | 1,69 | 0,⁠184 |

All the parameters analyzed in the NOR test before the sleep fragmentation protocol begins. The major significative changes are between wt and 5xFAD animals and not between the same strain. NF=not fragmented mice; F=fragmented mice

**Table S4: Differences in Y-maze data analyses between not fragmented (NF) and fragmented (F) animals before the beginning of the protocol.**

| **Y MAZE TEST** | | | | | | |
| --- | --- | --- | --- | --- | --- | --- |
| **PARAMETERS** | **NF wt** | **F wt** | **NF 5xFAD** | **F 5xFAD** | **ANOVA** | |
|  |  |  |  |  | **F** | **p-value** |
| **Alternation (%)** | 16,09 ± 1,07 | 14,90 ± 1,24 | 15,75 ± 1,43 | 20,40 ± 1,59 | 0,⁠22 | 0,⁠804 |
| **Max alternation (%)** | 28,45 ± 2,71 | 25,90 ± 2,07 | 27,92 ± 1,94 | 33,80 ± 2,18 | 0,⁠33 | 0,⁠719 |
| **Direct rivisit (%)** | 1,45 ± 0,⁠51 | 1,00 ± 0,⁠39 | 1,42 ± 0,7⁠40 | 0,⁠70 ± 0,⁠21 | 0,⁠31 | 0,⁠734 |
| **Indirect rivisit (%)** | 9,45 ± 1,38 | 9,00 ± 1,24 | 9,50 ± 0,⁠75 | 10,10 ± 1,24 | 0,⁠06 | 0,⁠945 |
| **Total frequency of entry into the arms (n°)** | 38,54 ± 2,83 | 32,80 ± 2,05 | 35,50 ± 2,46 | 42,70 ± 2,62 | 1,83 | 0,⁠179 |
| **Frequency of entry in arm 1 (n°)** | 11,90 ± 1,29 | 11,60 ± 1,01 | 12,25 ± 0,82 | 11,90 ± 1,60 | 0,⁠10 | 0,⁠909 |
| **Cumulative duration in arm 1 (s)** | 97,01 ± 7,67 | 109,28 ± 6,65 | 102,63 ± 5,67 | 100,04 ± 10,32 | 0,⁠81 | 0,⁠456 |
| **Frequency of entry in arm 2 (n°)** | 12,55 ± 1,27 | 10,70 ± 1,16 | 12,08 ± 1,15 | 14,80 ± 1,37 | 0,⁠60 | 0,⁠553 |
| **Cumulative duration in arm 2 (s)** | 91,30 ± 6,13 | 107,15 ± 10,24 | 88,21 ± 9,80 | 86,30 ± 9,00 | 1,24 | 0,⁠302 |
| **Frequency of entry in arm 3 (new) (n°)** | 14,09 ± 1,04 | 10,50 ± 0,⁠82 | 14,67 ± 1,27 | 12,60 ± 1,39 | 4,11 | **0,⁠026** |
| **Cumulative duration in arm 3 (new) (s)** | 125,20 ± 8,33 | 105,67 ± 7,69 | 122,80 ± 10,83 | 124,56 ± 15,37 | 1,25 | 0,⁠302 |
| **Latency to enter in arm 3 (new) (s)** | 5,15 ± 2,22 | 11,31 ± 3,18 | 7,49 ± 2,80 | 10,86 ± 6,71 | 1,21 | 0,⁠311 |
| **Frequency of entry in the center (n°)** | 34,80 ± 2,33 | 28,10 ± 1,97 | 35,50 ± 2,14 | 35,20 ± 1,95 | 3,39 | **0,⁠047** |
| **Cumulative duration in the center (s)** | 45,02 ± 3,74 | 36,88 ± 5,42 | 42,83 ± 3,06 | 37,02 ± 2,08 | 1,02 | 0,⁠373 |
| **Distance traveled in arena (cm)** | 4747,79 ± 3,74 | 1584,73 ± 91,20 | 1870,10 ± 115,83 | 2151,91 ± 121,43 | 1,23 | 0,⁠306 |
| **Frequency of protect rearing (n°)** | 44,10 ± 2,83 | 38,50 ± 3,34 | 38,73 ± 3,35 | 38,70 ± 4,23 | 0,⁠98 | 0,⁠387 |
| **Frequency of un-protect rearing (n°)** | 5,40 ± 1,18 | 5,90 ± 1,77 | 2,93 ± 1,01 | 2,60 ± 0,⁠56 | 1,51 | 0,⁠236 |
| **Frequency of grooming (n°)** | 2,80 ± 0,⁠18 | 2,50 ± 0,54 | 1,64 ± 0,⁠28 | 1,60 ± 0,⁠16 | 3,1 | 0,⁠060 |
| **Cumulative duration of grooming (s)** | 13,38 ± 2,64 | 18,41 ± 4,19 | 15,92 ± 6,40 | 11,60 ± 2,67 | 0,⁠26 | 0,⁠775 |

All the parameters analyzed in the Y-maze test before the sleep fragmentation protocol begins. Few significative changes are relevant between NF wt and F wt and between NF 5xFAD and F 5xFAD. NF=not fragmented mice; F=fragmented mice

**Figure S1: AQP4 and its isoform expression.**


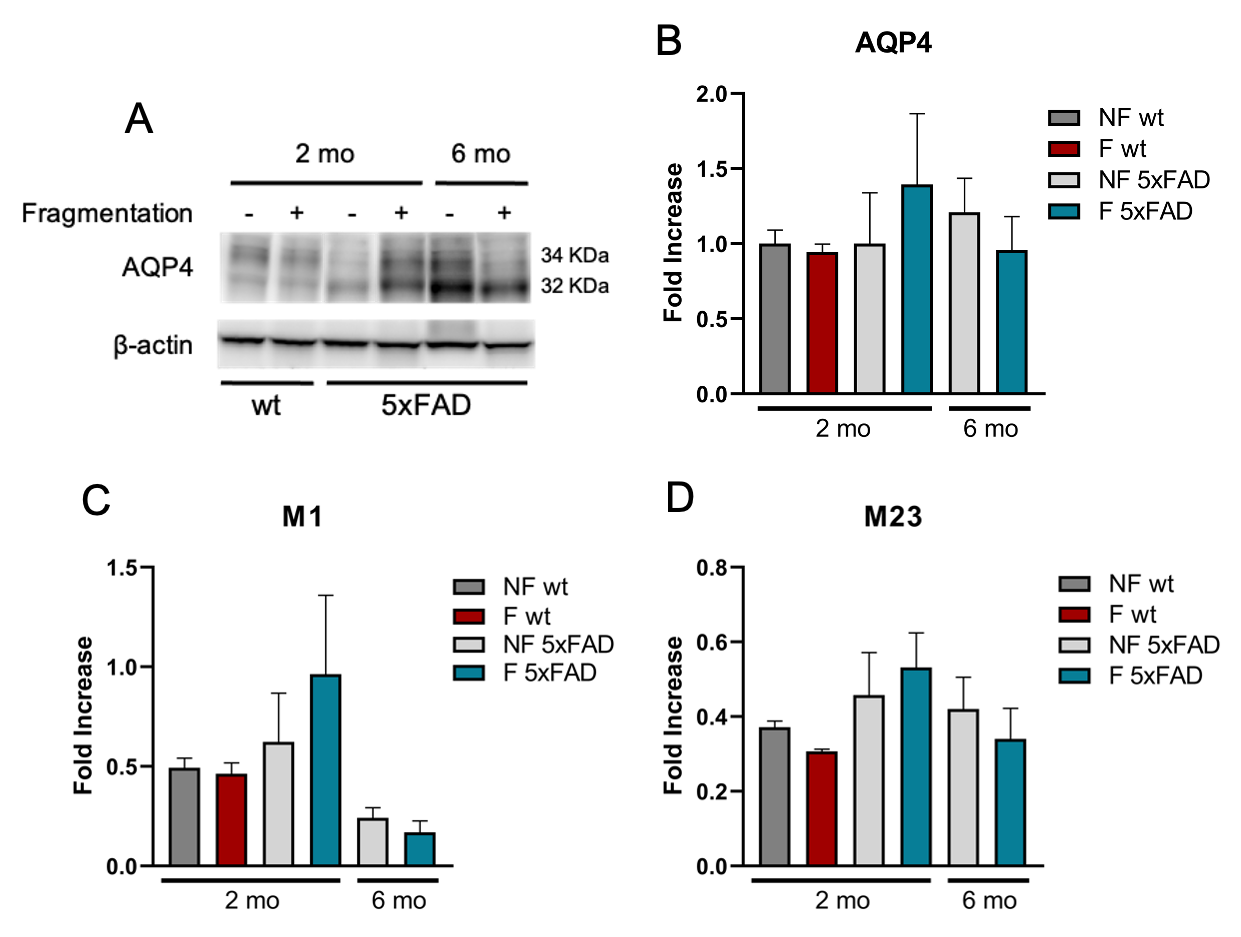


AQP4 and its isoform expression was evaluated with western blot analysis in both wt (2-month-old) and 5xFAD (2- and 6-month-old) mice. **A** Representative western blot of brain lysates from wt and 5xFAD mice of anti-AQP4 antibody. β-actin was used as loading control. **B** Densitometric analysis of total AQP4. **C** and **D** Densitometric analysis of the two main AQP4 isoforms: M1 (34 KDa) and M23 (32 KDa). NF=not fragmented mice; F=fragmented mice. The data are mean standard error of the mean (SEM) and they were analyzed by one-way ANOVA followed by Bonferroni *post-hoc* test, n=3 per condition


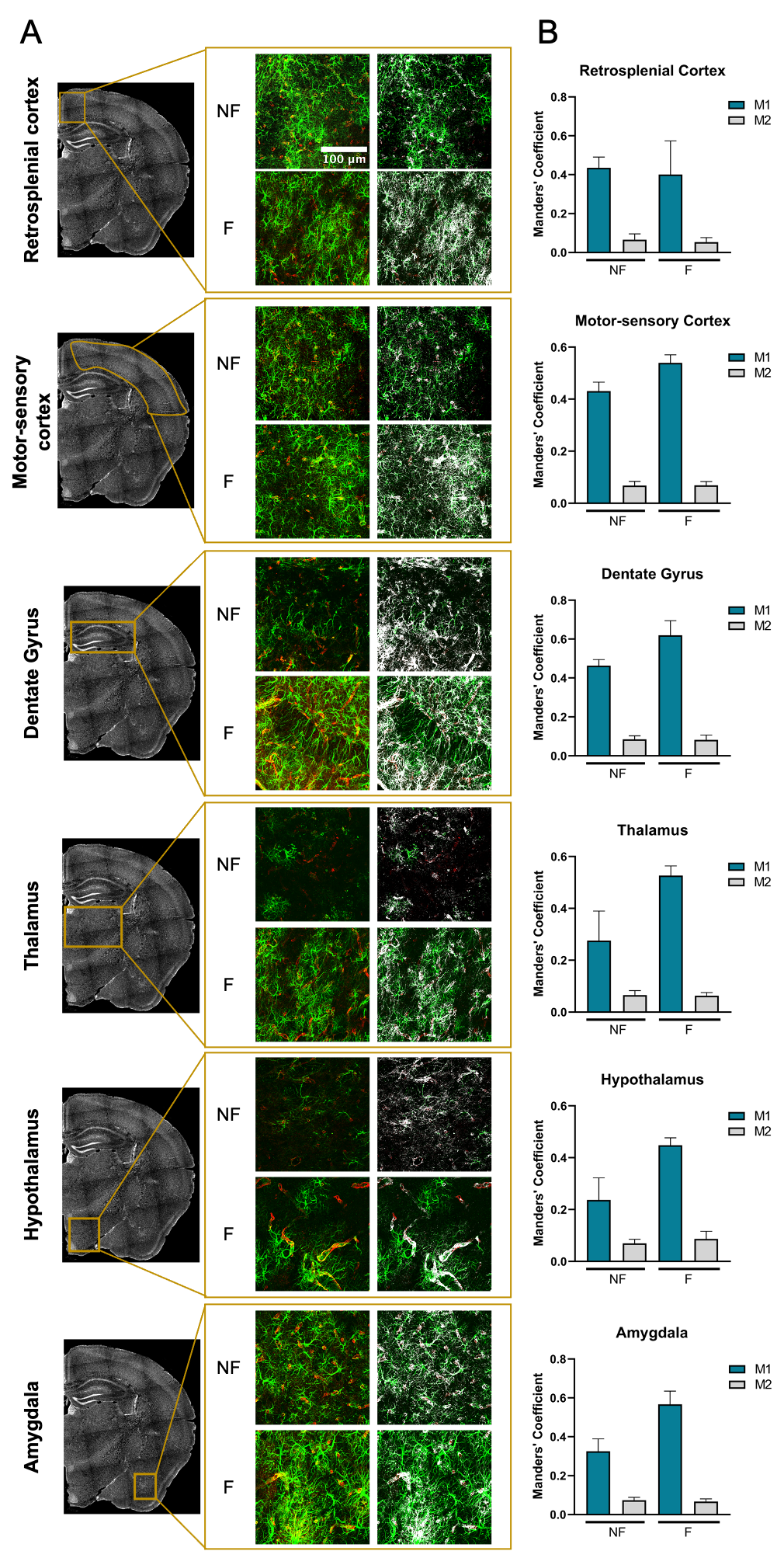
**Figure S2: AQP4 and GFAP colocalization analysis.**

AQP4 and GFAP colocalization analysis with JaCoP, a colocalization plugin of ImageJ. **A** Representative image of GFAP and AQP4 signal colocalization in the different brain areas. GFAP staining in green and AQP4 staining in red. On the right, the respective colocalization masks, with colocalizing pixels in white. **B** Histograms of colocalization analysis which was carried out with Manders’ coefficients M1 and M2. M1 represents AQP4-related pixels overlapping with GFAP signal, while M2 describes GFAP-related pixels overlapping with AQP4 signal. NF=not fragmented mice; F=fragmented mice. The data are mean standard error of the mean (SEM) and they were analyzed by one-way ANOVA followed by Bonferroni *post-hoc* test, n=3 per condition
